# Pooled CRISPRi screening of the cyanobacterium *Synechocystis* sp PCC 6803 for enhanced industrial phenotypes

**DOI:** 10.1038/s41467-020-15491-7

**Published:** 2020-04-03

**Authors:** Lun Yao, Kiyan Shabestary, Sara M. Björk, Johannes Asplund-Samuelsson, Haakan N. Joensson, Michael Jahn, Elton P. Hudson

**Affiliations:** 1 Science for Life Laboratory, KTH - Royal Institute of Technology, SE-171 21 Stockholm, Sweden; 2 Department of Protein Science, KTH - Royal Institute of Technology, SE-106 91 Stockholm, Sweden

**Keywords:** Industrial microbiology, Metabolic engineering, Bacterial systems biology, CRISPR-Cas systems

## Abstract

Cyanobacteria are model organisms for photosynthesis and are attractive for biotechnology applications. To aid investigation of genotype-phenotype relationships in cyanobacteria, we develop an inducible CRISPRi gene repression library in *Synechocystis* sp. PCC 6803, where we aim to target all genes for repression. We track the growth of all library members in multiple conditions and estimate gene fitness. The library reveals several clones with increased growth rates, and these have a common upregulation of genes related to cyclic electron flow. We challenge the library with 0.1 M L-lactate and find that repression of peroxiredoxin *bcp2* increases growth rate by 49%. Transforming the library into an L-lactate-secreting *Synechocystis* strain and sorting top lactate producers enriches clones with sgRNAs targeting nutrient assimilation, central carbon metabolism, and cyclic electron flow. In many examples, productivity can be enhanced by repression of essential genes, which are difficult to access by transposon insertion.

## Abstract (web summary)

Developing cyanobacteria as CO2-neutral cell factories relies on the knowledge of the regulation mechanisms for growth and metabolism. Here, the authors develop an inducible CRISPRi gene repression library in *Synechocystis* sp. PCC 6803 and screens genes potentially affecting growth and L-lactate tolerance and production.

## Introduction

Cyanobacteria are model organisms for photosynthetic electron flow, photorespiration, and the circadian clock^1–3^. In addition to their massive ecological importance, biotechnological applications of cyanobacteria have been proposed, such as microbial cell factories, where metabolism is engineered to synthesize chemicals from CO_2_ using energy derived from light^4–6^. This widespread interest in cyanobacteria brings a need for a system-wide analysis of gene essentiality, as well as tools for elucidating gene function. Even in model cyanobacteria such as *Synechocystis*, approximately 45% of genes have no assigned function (Cyanobase). Historically, the transposon mutagenesis library has been used to identify loss-of-function or gain-of-function mutants in bacteria. Variants of transposon mutagenesis tag the transposon insertion site with a barcode, which enables tracking and quantification of mutant growth via next-generation sequencing (NGS). Rapid screening of libraries in multiple growth conditions is one avenue to elucidating gene function^7^. A transposon library in the cyanobacterium *Synechococcus elongatus* PCC 7942 (hereafter *Synechococcus* PCC 7942) was used to map essential genes in both constant light and diurnal growth (12 h light, 12 h dark)^8,9^.

An alternative to transposon mutant libraries are pooled CRISPRi libraries for targeted gene repression, where unique single-guide RNA (sgRNA) genes are pooled and transformed into the strain of interest. Since the protospacer region of sgRNAs is small enough (~20 nt) to be sequenced by NGS, it can serve as a barcode and allows monitoring of the abundance of each clone in the library. Important for phenotyping, inducible CRISPRi allows modulation of essential genes. CRISPRi libraries have been applied to screen gene essentiality and diverse phenotypes in various model bacteria, including morphology, solvent tolerance, and phage resistance^10–12^. Inducible CRISPRi has been developed for several cyanobacteria strains^13,14^, but pooled sgRNA libraries have not been exploited.

**Fig. 1:**
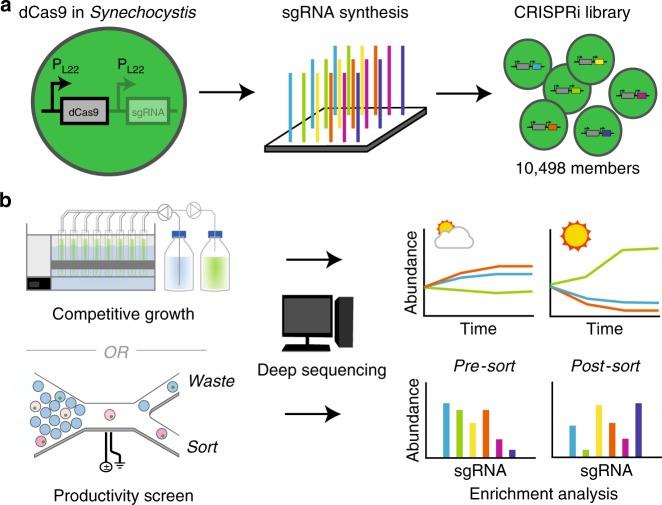
Workflow for CRISPRi screening in *Synechocystis*. **a** Creating the CRISPRi library. An inducible promoter P_L22_ is used in *Synechocystis*. **b** The fitness or productivity of each library member is assessed by counting mutants in a bioreactor cultivation or in a sorted population using next-generation sequencing.

Here we report the construction and use of an inducible CRISPRi library of 10498 clones for the model cyanobacterium *Synechocystis* sp. PCC 6803 (hereafter *Synechocystis*). Each clone expresses dCas9 and an sgRNA that targets a protein-coding open reading frame (ORF) or a non-coding RNA (ncRNA). We track the composition of the library during growth in multiple conditions, including different light regimes and in the presence of the organic acid L-lactate. We also screen the library for enhanced L-lactate production using droplet microfluidics (Fig. 1). In addition to providing fitness scores for almost all ORFs and ncRNAs in *Synechocystis*, our results give insights into how to engineer cyanobacteria metabolism for industrial use. We show that there are multiple gene knockdowns that increase the growth rate of *Synechocystis*, though these genes regulate similar processes in photosynthesis. We find far fewer diurnal-specific fitness genes in *Synechocystis* when compared to previously published data from *Synechococcus* PCC 7942. Further, we provide support for previous computational predictions that alteration of the ATP/NADPH balance can improve bioproduction in cyanobacteria. By screening both growth and productivity, this platform can yield mutants that trade biomass formation for increased productivity of a target compound. While we have focused on biotechnological traits of interest here, the CRISPRi library can be used to explore the connection between growth and robustness in many conditions, all while allowing access to modulation of essential genes. The data for all competition experiments performed with the sgRNA library can be accessed through an interactive web application [https://m-jahn.shinyapps.io/ShinyLib/].

## Results

### Gene fitness during photoautotrophic growth

We designed a CRISPRi library for repression of each annotated gene in *Synechocystis* (3546 ORFs, 1871 ncRNAs)^15^. We designed two sgRNAs for each of 3526 ORFs and 1555 ncRNAs, and one sgRNA for 20 ORFs and 316 ncRNAs, totalling 10498 sgRNAs (Methods, Supplementary Data 1, Supplementary Data 2). The resulting 10498 sgRNA sequences were synthesized, pooled, and cloned into a genomic integration vector, and the pooled sgRNAs were transformed into a *Synechocystis* strain containing a genome-integrated, anhydrotetracycline (aTc)-inducible dCas9 cassette. The resulting *Synechocystis* clones were pooled and constituted the CRISPRi library. Deep sequencing of the sgRNA region of the library confirmed that all of the designed 10498 sgRNAs were present.

**Fig. 2:**
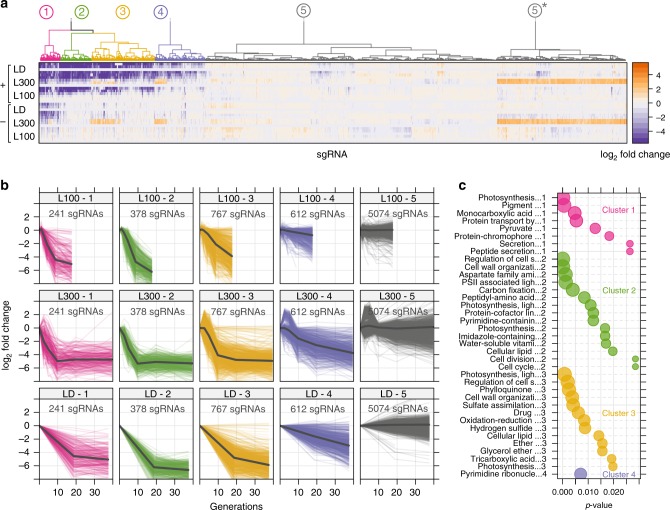
Dynamics of CRISPRi library during photoautotrophic growth. **a** Clustered sgRNAs by similarity of log_2_ fold-change over time. Rows represent time-points sampled after induction. (L100—light with 100 µmol m^−2^ s^−1^, L300—light with 300 µmol m^−2^ s^−1^, LD—light-dark cycle). All log_2_ fold-change values were calculated from averages of four replicate cultivations. Symbols: plus, induced, minus, non-induced, star, cluster 5 is a combination of two unchanged clusters. **b** Log_2_ fold-change of individual sgRNAs over the course of each cultivation. Experiment run time was normalized to number of cell generations estimated from population growth rate. **c** Enriched gene-ontology (GO) terms for the four clusters (1–4) showing sgRNA depletion; *p*-value—Fisher’s exact test with elimination (see Methods). Source data are provided as a Source Data file.

We cultivated the library in light-limited turbidostats under two constant-light conditions, 100 μmol photons m^−2^ s^−1^ (L100) and 300 µmol photons m^−2^ s^−1^ (L300) and a light-dark diurnal condition (LD, sinusoidal illumination up to 300 µmol photons m^−2^ s^−1^ over a 12 h period followed by 12 h darkness), each with supplemented 1% CO_2_ and in four replicates. Samples for NGS were taken periodically over 32 cell generations (L100 and LD cultivations were 32 days, µ ~0.03 h^−1^; L300 cultivations were 16 days, µ ~0.07 h^−1^; Supplementary Data 3). For each sampling point, the abundance of the 7072 sgRNAs targeting ORFs were quantified and averaged across the four replicates. The sgRNAs were grouped into five clusters based on their rate of washout (depletion) from the turbidostat (Fig. 2a). In total, 1998 sgRNAs (28.3%) had a significant depletion in at least one condition, indicating these target genes have some contribution to cell fitness (Fig. 2b). Cluster 1 (241 sgRNAs) contained sgRNAs that were depleted in induced and uninduced cultivations, revealing weak background leakage of dCas9 and particular sensitivity to changes in abundance of these genes. Cluster 2 (378 sgRNAs) was enriched in sgRNAs that were quickly depleted in all growth conditions. Cluster 3 (767 sgRNAs) contained sgRNAs that were more rapidly depleted in L300 than L100. Cluster 4 (612 sgRNAs) contained sgRNAs that were depleted slowly, and cluster 5 (5074 sgRNAs) contained sgRNAs that were not depleted at all. Cluster 5 combines two very similar clusters differing only in the temporary enrichment of a subset of sgRNAs, most likely due to technical variation (L300, 1 and 2 d time-points). A gene-ontology (GO term) enrichment analysis showed that clusters 1 and 2 were highly enriched for GO terms related to core cellular processes such as photosynthesis, carbon fixation, and translation (Fig. 2c). Cyanobacteria invest most of their resources into synthesizing proteins from these groups^16,17^. Interestingly, nearly all sgRNAs with a low fitness score (i.e., they are depleted from the library) targeted genes whose expression were found by Jahn et al. to be regulated with growth rate (Supplementary Fig. 1, protein abundance based on mass spectrometry measurements)^16^. Cluster 3 was enriched for a more diverse set of GO terms (e.g., secondary metabolites, cell membrane) and cluster 4 was enriched in only one GO term (nucleotide metabolism).

Fitness scores for genes in central carbon metabolism can be used to refine flux predictions of core metabolic models. Here, data revealed instances of both rigidity and plasticity in *Synechocystis* central carbon metabolism. For example, the production of pyruvate in *Synechocystis* has been predicted to be mainly through decarboxylation of oxaloacetate by NADP-dependent malic enzyme, not dephosphorylation of phosphoenolpyruvate by pyruvate kinase^18^. However, malic enzyme (*me, slr0721*) and pyruvate kinase (*pyk2*, *sll1275*) both have low fitness scores (Supplementary Fig. 2). This suggests that one route to pyruvate cannot fully substitute for the loss of the other, though interpretation of *pyk2* fitness is complicated by its position in an operon (see Discussion). Within the Calvin cycle, two energetically equivalent routes for synthesis of fructose-6-phosphate have been considered: through class-I fructose-bisphosphate aldolase (*fda, slr0943*) or through transaldolase (*talB, slr1793*)^19^. Neither of these genes had a low fitness score, though the class-II FBP/SBP aldolase gene (*cbbA, sll0018)*, which is needed to synthesize sedoheptulose bisphosphate for both proposed routes, did have a low fitness score. While these results do not definitively show which route is favored under photoautotrophic conditions, they suggest that flux can operate through either route if the other is perturbed. This flexibility is in contrast to *Synechococcus* PCC 7942, where a transposon insertion library showed that the sole FBP aldolase gene was essential for photoautotrophic growth, and transaldolase was not^8^.

**Fig. 3:**
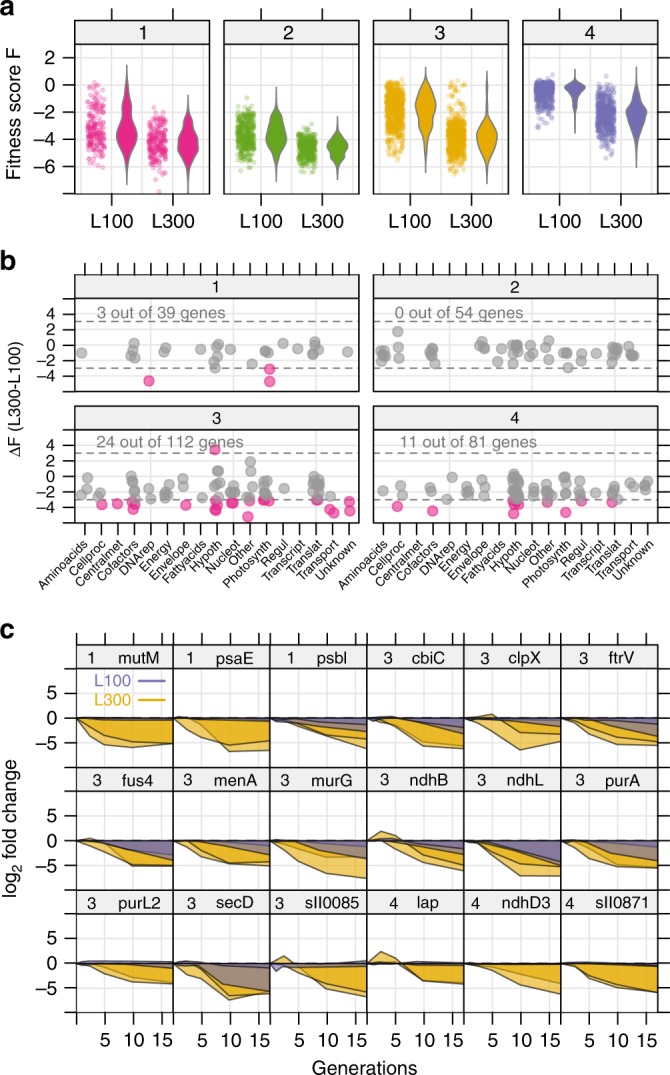
Genes with condition-dependent fitness in two light conditions. **a** Distribution of fitness score *F* for all sgRNAs in clusters 1–4 (color code as in Fig. 2). Fitness score indicates the degree of enrichment (positive) or depletion (negative) of an sgRNA, normalized to cell generations. Comparison between two light conditions (L100—light with 100 µmol m^−2^ s^−1^ and L300—light with 300 µmol m^−2^ s^−1^) shows sgRNAs are on average more rapidly depleted under L300 independent of number of cell generations. **b** Difference in fitness (Δ*F*) between L100 and L300, for genes with both sgRNAs in the same cluster. Differentially depleted/enriched sgRNAs indicated in red, threshold: 3 ≤ ΔF ≤ −3. **c** Time courses of both sgRNAs for a selection of 18 genes with high Δ*F* in L100 (blue) and L300 (yellow). Source data are provided as a Source Data file.

We next compared fitness scores for sgRNAs in each growth condition (see Methods for calculation). Fitness scores were generally lower in the L300 condition than in L100, even when normalized to number of cell generations (Fig. 3a). One explanation could be a higher protein turnover rate in the higher growth condition. Lower fitness scores at L300 could also be expected for genes mediating high light or redox stress acclimation. In order to find genes important for specific light conditions, we calculated gene fitness as the mean fitness of two sgRNAs, and selected only genes where both sgRNAs were present in the same cluster. We found 38 genes that were beneficial for growth in L300 but neutral in L100, according to a difference in gene fitness ≥ 3 (Fig. 3b). This set was almost exclusively in cluster 3 and 4 and included genes related to DNA repair, proteome and redox homeostasis, and subunits of PSI and the NADPH dehydrogenase NDH-1 (Fig. 3c). Energy dissipation and photo-stress response are thus particularly important for growth at high light. There were 22 genes with differential fitness between L100 and LD, and only 5 between L300 and LD (Supplementary Fig. 3 and 4). The extensive overlap between L300 and LD suggests that the LD condition may involve light stress, likely at dawn. The five genes with different fitness scores between L300 and LD include two CO_2_ hydration genes (*ndh3, slr1302*). The dearth of genes with LD-specific fitness contributions is in contrast to *Synechococcus* PCC 7942, where more than 100 genes were found with a transposon library to be important for LD growth^9^. This discrepancy could be attributed to the CRISPRi library comprising partial repressions and not gene knockouts. Furthermore, *Synechococcus* has a more pronounced day-night rhythm than *Synechocystis*, with a higher fraction of oscillatory transcripts and larger amplitudes^20^.

### Mutants with a growth advantage

**Fig. 4:**
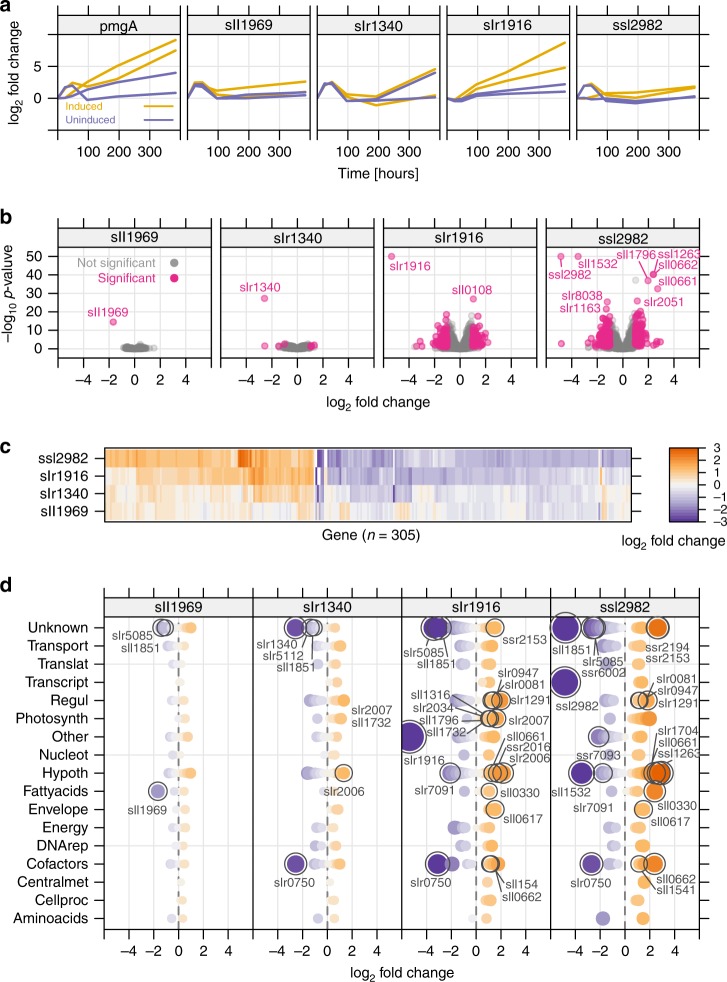
Transcriptomics of faster-growing mutants. **a** Enrichment of faster-growing sgRNA mutants in library competition (turbidostat) experiment at L300 (300 µmol photons m^−2^ s^−1^), log_2_ fold-change of read count over time (*n* = 2 sgRNAs). **b** Volcano plot representation of transcriptomics data from four reconstructed sgRNA mutants. Shows log_2_ fold-change for gene expression compared to the control strain (sgRNA-NT0), against adjusted *p*-value for each gene. Gray—non-significantly different genes, pink—significantly different genes (threshold: negative log_10_ *p*-value ≥ 2; absolute log_2_ fold-change ≥ 1). The *p*-value for three sgRNA mutants was outside the plotting region and was restricted to −log_10_ of 50 for visibility. **c** Heat map representation of transcriptomics data from four reconstructed sgRNA mutants. Shows all 305 genes that were significantly different in at least one of the four mutants, clustered into two different groups based on similarity of gene expression. **d** Significantly different genes are sorted by Cyanobase pathways. Genes of particular interest were highlighted (see text for details). Blue and orange indicates negative and positive log_2_ fold-change, respectively. Size of symbols increases with increasing absolute change. Source data underlying Fig. 4b, d are provided as a Source Data file.

Quantification of the library population over time in a turbidostat allows calculation of the maximum specific growth rate µ of each mutant (Methods). Growth-rate estimates allow for more intuitive interpretation of mutant fitness and highlights that library clones are repression mutants, not total knockouts. For example, repression of Calvin cycle genes often caused significant reduction of µ (e.g*., prk* −40%, *cbbA* −95%), while repression of most photorespiration-related genes did not (e.g*., glcD1* −10%, *glcD2* −10%; Supplementary Fig. 2). Many clones showed higher growth rates than the population average, notably those targeting *pmgA (*mean increase for L100 and L300 + 17%) and *slr1916* (mean increase + 13%). The *pmgA* and *slr1916* repression clones also showed higher growth rates in the diurnal condition (Fig. 4a). PmgA is a regulator involved in the high-light response in *Synechocystis* and a *pmgA* knockout mutant has an inability to reduce PSI content in high light, resulting in more efficient photosynthesis^21^. A *slr1916* mutant was previously identified from a small *Synechocystis* transposon library on the basis of altered fluorescence kinetics and also shows a higher PSI content at high light^22^. Notably, other mutants isolated in the study of Ozaki et al. were not enriched in our library, indicating that altered PSI/PSII alone does not ensure faster growth^22^. *Slr1916* was annotated based on homology as *menH*, an esterase in the phylloquinone pathway. However, repression of the 8 other phylloquinone pathway genes resulted in strong growth defects, suggesting *slr1916* is not a key enzyme in this pathway (Supplementary Fig. 5). Furthermore, *slr1916* is localized to the plasma membrane^23^, though its proposed substrates are soluble. Three additional sgRNA clones showed slight but significant increases in growth rate in the turbidostat data (+5%): *sll1969*, an annotated triacylglycerol lipase in the same operon as *pmgA; slr1340*, an uncharacterized gene encoding a predicted acetyl-transferase, and *ssl2982*, encoding the non-essential ω subunit of RNA polymerase^24^.

In an attempt to uncover common regulations among these mutants, we reconstructed axenic knockdown strains. The repression clones of *pmgA*, *slr1916, sll1969*, and *ssl2982* each had a higher maximum growth rate than the control strain, as measured in the exponential phase of batch cultivation, while the growth rate of the *slr1340* repression clone was not higher than the control strain (Supplementary Fig. 6). We next collected transcriptomics of strains after CRISPRi induction. A *pmgA* clone was not included as microarray data from a *pmgA* knockout was reported previously^25^. From the RNA-Seq data, *slr1916* and *ssl2982* mutants had 143 and 248 differentially expressed genes compared to a control strain (sgRNA-NT0, with no target site in *Synechocystis* genome) (Fig. 4b). A weak transcriptomic response of *slr1340* and *sll1969* mutants could be due to a lower repression efficiency in these clones (log_2_ FC of target genes was −2.57 and −1.67, for *slr1340* and *sll1969*, respectively, compared to −5.37 and −4.85 for *slr1916* and *ssl2982*). Clustering genes based on similarity of expression changes revealed that the same set of genes was affected for all mutants, but with stronger effects in *slr1916* and *ssl2982* (Fig. 4c). The high-light responsive transcription factor RpaB (*slr0947*, regulator of phycobilisome association B) was upregulated in multiple mutants. The extensive regulon of RpaB includes likely repressor activity of linear electron transport at high light (e.g., subunits of PSII and Cyt-b_6_f), and activation activity of photoprotection and cyclic electron flow (e.g., *ftsH* and *ssr2016*)^26^. Other common upregulated genes are involved in electron transport (*fed7*), and include components of the NDH-1 complex *(ndhD1, ndhD2*) and carotenoid biosynthesis (*crtQ* and *crtZ)* (Fig. 4d). Cyanobacterial NDH-1 complexes participate in cyclic electron flow around PSI, respiratory electron flow, and CO_2_ uptake^27^. Genes that were downregulated in all four mutants were *sll1851*, a small non-annotated gene, and *chlN* (*slr0750*), a subunit of the light-independent operative protochlorophyllide oxidoreductase (LI-POR).

### Gene fitness in the presence of L-lactate

**Fig. 5:**
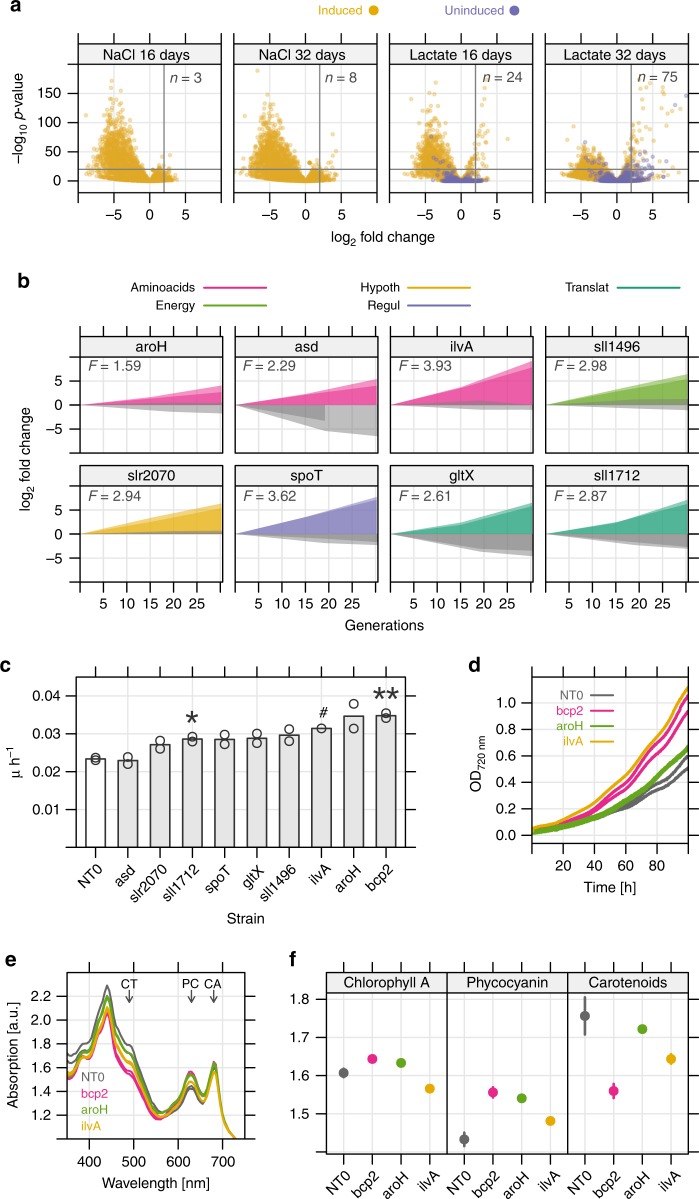
SgRNA clones with improved L-lactate tolerance. **a** Volcano plot showing enrichment of 75 sgRNAs during cultivation with added 0.1 M sodium L-lactate after 32 d, (threshold: log_2_ fold-change ≥ 2, −log_10_ *p*-value ≥ 20). Only 8 sgRNAs were enriched in a 0.1 M NaCl control cultivation. **b** Eight genes where both sgRNAs were enriched above average in 0.1 M L-lactate cultivation (coloured) but not NaCl cultivation (gray). Color indicates association to a cyanobase pathway. *F* - mean fitness score of two sgRNAs. **c** Mean growth rate µ (h^−1^) of selected, reconstructed knockdown strains (*n* = 2 independent replicate cultivations) over the first 80 h of batch cultivation with L-lactate added to 0.1 M. Symbols show significance between the control strain (NT0) and mutants using 1-sided student’s *t*-test. *p-value ≤ 0.05, **p-value ≤ 0.01 (sll1712, *p* = 0.019; bcp2, *p* = 0.006), #—only 1 replicate was used for *ilvA* mutant. **d** Example of growth advantage in batch culture with L-lactate added to 0.1 M of sgRNA clones *bcp2*, *aroH*, and *ilvA*. **e** Absorption spectra of fastest-growing sgRNA mutants and NT0 in 0.1 M L-lactate. Arrows mark absorption maxima for pigments. CT carotenoids, PC phycocyanin, CA chlorophyll A. **f** Relative pigment absorption for carotenoids, phycocyanin, and chlorophyll A for the selected strains with L-lactate added to 0.1 M. Source data underlying Fig. 5a, b are provided as a Source Data file.

The CRISPRi library is also beneficial for finding stress tolerance phenotypes, which are generally difficult to engineer rationally. The commodity chemical L-lactate has been produced in several cyanobacteria but still at relatively low titers (up to 15 mM)^28^. The tolerance of *Synechocystis* to L-lactate is ~0.1 M (9 g/L), at which specific growth rate is reduced by 50% (L100 condition, Supplementary Data 3). To identify mutants with increased tolerance to L-lactate, we cultivated the library in turbidostats with added 0.1 M sodium L-lactate (pH-adjusted) and sampled periodically for NGS (0, 16, and 32 d). We found 75 sgRNAs that were enriched during the L-lactate cultivation, but not in a NaCl control cultivation (Fig. 5a). Eight genes were enriched with both sgRNAs (Fig. 5b). Curiously, 19 of the enriched sgRNAs (24%) targeted genes in amino-acid metabolism and protein biosynthesis, including multiple amino-acid tRNA synthetases (*argS, aspS, asnS, glyS, gltX, metS*) (Supplementary Fig. 7). Many of these clones had growth defects in the absence of L-lactate (NaCl control, grey area in Supplementary Fig. 6), which supports the general phenomenon that slow-growing microbes are more stress tolerant^29^.

We reconstructed nine of the repression clones to confirm growth improvement in batch cultures with added L-lactate (Fig. 5c, d, Supplementary Fig. 8). Of the nine clones, the *sll1712* and *bcp2* mutants had significantly higher growth rates than the control strain (Fig. 5c). The *bcp2* mutant (bacterioferritin co-migratory protein, a peroxiredoxin) had the largest improvement, a 49% increase in µ (*p* = 0.006, student’s *t*-test). We tested L-lactate consumption of all mutants and found that none consumed L-lactate over a 48-hour period. The mechanism for increased tolerance in the *bcp2* mutant is not known. However, thioredoxins can mediate direct reduction of cysteines on transcription factors, including the master RpaB regulator in *Synechocystis*^30^. The absorbance spectra of all L-lactate-tolerant mutants showed increased chlorophyll A and phycocyanin absorption in the presence of L-lactate (Fig. 5e, f). Relative concentration of carotenoids, pigments related to the light stress response, was reduced in L-lactate tolerant mutants.

We could also identify clones with growth negatively affected by L-lactate but not by NaCl (Supplementary Fig. 9). These targeted genes could thus be candidates for overexpression to improve tolerance. Most prominent were an antibiotic resistance gene (*zam, sll1910*), two nucleases, the protease *clpX* (*sll0535*), cytochrome M (*cytM, sll1245*) that may act to dissipate excess electrons^31^, and *sepF* (*slr2073*), an inhibitor of cell division.

### Screening the CRISPRi library for L-lactate productivity

The CRISPRi library can be linked to screens other than growth, and we next sought to find mutant clones that had increased productivity of L-lactate. The sgRNA pool and inducible dCas9 cassette were cloned into a *Synechocystis* strain containing lactate dehydrogenase from *Lactococcus lactis*^32^. The resulting L-lactate CRISPRi library was characterized by NGS and contained 10494 unique sgRNA clones. To screen the library for a secreted product, we used droplet encapsulation and microfluidics sorting^33,34^. First, the L-lactate CRISPRi library was grown in shake-flasks, and gene repression was induced by addition of aTc. Cell aliquots were taken after 36 h and 66 h to assay for L-lactate productivity (Methods, Supplementary Fig. 10). The productivity assay involved encapsulation of cells in droplets, followed by a picoinjection of each droplet with the components of a fluorescent L-lactate assay. In each droplet, secreted L-lactate reacts with the assay, allowing droplets to be sorted based on fluorescence. The cultivation and two-time point productivity assays were done on two separate occasions, resulting in four sorting runs. For each run, approximately 180,000 cell-containing droplets were screened and 36,000 droplets were sorted. Cells could not be reliably recovered on agar plates after sorting, so we performed PCR of the sgRNA region directly from sorted droplets, followed by NGS library preparation and quantification. In each sorted sample, 1500–5000 unique clones were detected with confidence (>32 reads; Methods) (Supplementary Data 4). Due to the small number of sorted cells and high variability between replicates, it was not possible to determine the significance of enrichment for each clone in the sorted populations. To assess which clones produced more L-lactate than average, we used instead the criteria that a clone had to be at least 3-times more abundant in the sorted fraction relative to the unsorted fraction. This criterium returned between 500 to 900 clones per sorted sample. Further filtering of clones enriched in at least two of the four sorted samples returned 397 clones, 266 harbored sgRNAs targeting ORFs and 131 harbored sgRNAs targeted to ncRNAs. Twenty-three clones were enriched in three of the four samples, and one clone was enriched in all four samples.

Though nearly half of the enriched clones targeted genes of unknown function, it is possible to derive engineering strategies from the annotated targets (Table 1). Alterations in carbon flux in cyanobacteria can be achieved by restriction of nutrient uptake or assimilation^35–37^. Enriched targets in nutrient uptake include glutamate dehydrogenase (*gdhA*), glutamate synthase (*gltB*), and glutamine synthase (*glnA)*, repression of these would restrict NH_3_ assimilation, as well as a nitrogen transporter *(nrtD2*) and a phosphorous transporter (*pstC*). Direct alteration of carbon flux was also apparent among enriched clones. For example, repression of 3-phosphoglycerate dehydrogenase (*serA*), citrate synthase (*gltA)*, and phosphoketolase (*slr0453)* could each be expected to increase pools of pyruvate, the precursor to L-lactate^31,38,39^. A second engineering strategy is suggested by target genes in electron transport and energy metabolism. Computational^40,41^ and experimental^42,43^ studies have shown that efficient production of some biochemicals would require lowering the ATP/NADPH ratio in the cyanobacteria cell. The enriched targets *ssr2016* and *ndhD2* are both involved in ATP-generating cyclic electron flow around PSI in *Synechocystis*^44^. *SdhB* is a subunit of succinate dehydrogenase contributing to respiration. The flavodiiron protein *flv3* (*sll0550*) catalyzes photoreduction of O_2_ during stress conditions. Knockout of *flv3* was shown to lower cellular ATP/NADPH ratio in *Synechocystis*^43^. Finally, the CCA-tRNA nucleotidyltransferase *pcnB*, essential for tRNA maturation and thus protein synthesis, was also enriched.

**Table 1:** Selection of sgRNA clones enriched by droplet sorting.

sgRNA	Appearances	Effect on growth	Gene function
gdhA_25*	4	None	Glutamate dehydrogenase
ssr2016_62*	3	Impaired	Ferredoxin:plastoquinone reductase, cyclic electron flow
gltA_41*	3	Impaired	Citrate synthase
pntB_19	3	None	Transhydrogenase subunit
pcnB_64*	3	Impaired	Nucleotidyltransferase
serA_10*	2	Impaired	Phosphoglycerate dehydrogenase
gltB_3*	2	Impaired	Glutamate synthase
ndhD2_15	2	None	NDH-1 complex subunit, cyclic electron flow
sdhB_26*	2	None	Succinate dehydrogenase
Entry85_13	2	None	atpA (ATP synthase) asRNA
pstC_41	2	None	Phosphate transporter
slr0453_10	2	None	Phosphoketolase

Note: Mutants were identified from a CRISPRi library based on L-lactate productivity. Appearance is the number of sorting runs (out of four) where that clone had an enrichment factor of at least 2 (normalized abundance in sorted population/normalized abundance in unsorted population). Clones where growth was impaired are denoted. These clones were significantly depleted in the L300 dataset after four generations (*ssr2016_62*, p_adj_ = 1.6e^−94^; *gltA_41*, p_adj_ = 3.1e^−53^; *pcnB_64*, p_adj_ = 1.5e^−58^; *serA_10*, p_adj_ = 3.6e^−21^; *gltB_3*, p_adj_ = 0.015; multiple-hypothesis adjusted *p*-values calculated by DESeq2). An asterisk denotes sgRNA mutants selected for reconstruction and validation. Source data are provided as a Source Data file.

**Fig. 6:**
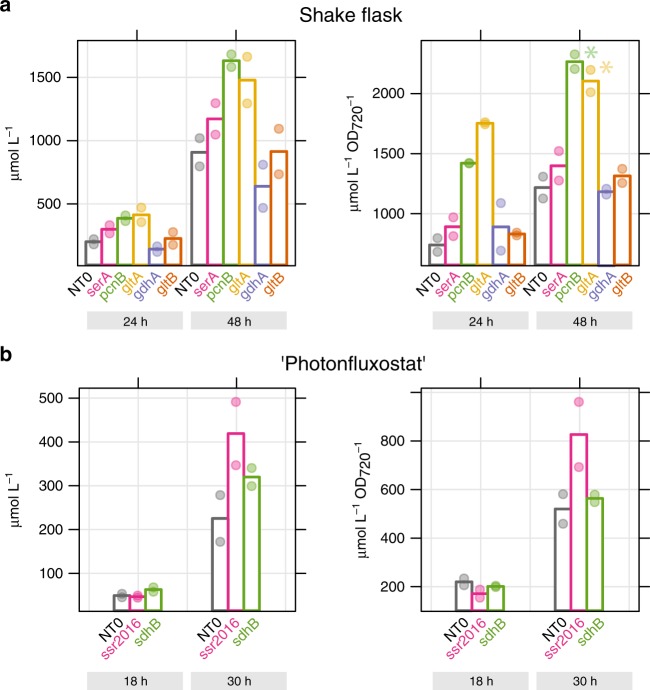
SgRNA clones with increased L-lactate production. Mutants with potentially increased L-lactate productivity were discovered using a fluorescence-activated droplet sorting assay. Seven selected mutants were reconstructed, cultivated, and product titer determined in µmol per liter, and µmol per liter per biomass (OD_720 nm_). **a** Productivity of selected mutants cultivated in axenic shake-flask cultures (n = 2, each strain assayed in two independent replicates). L-lactate concentration was measured after 24 and 48 h. NT0 - control sgRNA with no target site in *Synechocystis* genome. *—significant with *p* ≤ 0.05, two-sided student’s *t*-test, pcnB, *p* = 0.016, gltA, *p* = 0.021. Bars are average values and individual values are shown as points. **b** A subset of two mutants was cultivated in axenic ‘photonfluxostat’ cultures (light intensity proportional to cell density, 1000 µmol photons m^−2^ s^−1^ OD_720 nm_^−1^, *n* = 2, each strain assayed in 2 separate experiments). L-lactate concentration was measured after 18 and 30 h. Bars are average values and individual values are shown as points. Source data are provided as a Source Data file.

We reconstructed eight clones for validation of L-lactate productivity, representing targets within nutrient uptake, carbon flux, and redox and energy generation (Table 1). In a first screen, we cultivated clones in shake-flasks. Cultures were induced for gene repression 2 days prior to inoculation. Only the *gltA* and *pcnB* strains had a significantly higher L-lactate titer than the control strain (Fig. 6a). The effect was enhanced when titers were normalized to cell density, indicating a redirection of carbon flux from biomass formation to product^38^. Two of the potentially redox-altered mutants (*sdhB, ssr2016*) were tested in a photonfluxostat reactor, where light intensity was gradually increased with cell density to ensure a fixed light dosage per cell^45^. This cultivation mode was expected to activate alternative electron flow reactions for an extended time, so as to amplify any effects of repressing these on L-lactate productivity. In photonfluxostat mode, the *sdhB* and *ssr2016* clones again had higher L-lactate titers than the control strain, but variability was high and the effect was not statistically significant (Fig. 6b). We note that titers and specific productivities were lower for all strains in the photonfluxostat mode than in shake-flasks, which could be due to altered gas transfer or different perceived light intensities.

## Discussion

We have primarily used the CRISPRi repression library to identify potential mutants with improved industrial phenotypes, which we then validated with screening of individual clones. By providing fitness contributions of all genes in a certain condition, the CRISPRi library reveals patterns in how cyanobacteria could solve certain physiological challenges. There were multiple genetic avenues for enhancing the growth rate of *Synechocystis*, though they converged on a similar transcriptome that upregulates photoprotection and electron-transport around PSI. An increase in PSI activity at high light can dissipate pressure in the electron-transport chain; a similar phenomenon was reported to contribute to high-light tolerance and faster growth in *Synechococcus elongatus* UTEX 2973 compared to its close relative *Synechococcus* PCC 7942^46^. This is also in-line with a recent modeling study, which showed that the rate of ATP and NADPH generation exerted the most control over carbon fixation rates in *Synechocystis*^47^. Interestingly, four out of five sgRNA targets in top faster-growing strains are potentially regulator proteins (*pmgA*, *slr1916*, *ssl2982*, *slr1340*). This finding is reminiscent of the mutation in the master regulator RpaA of *Synechococcus elongatus* UTEX 2973 that drastically alters the transcriptome and is necessary for unlocking fast growth^48^. The RNAP omega subunit (*ssl2982*) is critical for acclimation of *Synechocystis* from low to high CO_2_^49^. That repression of *ssl2982* provides a growth advantage at high light and high CO_2_ is surprising. However, our experimental setup differs from that used previously, in that we perform a knockdown of *ssl2982*, and that cells are already pre-acclimated to high CO_2_ before repression.

Care should be taken when using results from CRISPRi libraries to determine gene essentiality. We used 2 sgRNAs targeting each ORF; recent studies have shown that more are needed to ensure statistical significance when assessing gene essentiality^12^. Furthermore, a confident determination of gene essentiality from CRISPRi data would require consideration of a gene’s place in an operon. Data from an *E. coli* CRISPRi library showed a strong polar effect, where targeting the first gene in an operon also represses downstream genes, while the reverse polar effect, i.e., repression of downstream genes affecting expression of upstream genes, was not prominent^50^. For *Synechocystis*, transcription start sites have been mapped in detail for several growth conditions and are annotated in the reference genome (NC_000911.1)^15^. Therefore, fitness data for individual genes can be considered with their operon context. For example, the *pyk2* gene *(sll1275)* encoding pyruvate kinase 2 is at the 5′ end of an operon that also contains *sll1276* (probable iron transporter) and *recF* downstream. In our data, both sgRNA clones targeting *pyk2* showed severe growth defects, suggesting it is important for cell growth. However, one sgRNA clone targeting *sll1276* also shows a strong growth defect. Considering previous attempts to knockout *sll1276* were unsuccessful^51^, it is likely that *sll1276* is essential for cell growth. Based on the sgRNA data alone, we cannot unambiguously conclude that *pyk2* is essential for growth, as the downstream *sll1276* is also likely repressed in those clones.

For ~50% of the genes with low fitness scores in our data, one sgRNA was significantly depleted from the library and the other was not (Supplementary Fig. 11). There are several lines of evidence which suggest that this discrepancy is due to weak binding of one of the sgRNAs (a false-negative) instead of off-target binding of an sgRNA to an essential gene elsewhere (false-positive). First, while repression efficiency of sgRNAs targeting near the start codon is typically >90%, it can be as low as 50%, so partial repression of an essential gene may not elicit a phenotype^13^. Second, 86% (283/329) of genes predicted to be essential by Flux Balance Analysis of a *Synechocystis* genome-scale model had at least one associated sgRNA clone significantly depleted^52^ (p_adj_ < 0.005; Supplementary Data 5). There is also a 75% agreement in our calculated fitness scores for genes in central carbon metabolism to their orthologs in *Synechococcus* PCC 7942, the latter determined by Tn-Seq^8^ (Supplementary Data 6). Third, off-target binding by dCas9 was shown to be problematic for strongly expressed dCas9^50^, while our genome-integrated *dCas9* was driven by a weak promoter. However, dCas9 is potent, as we observed gene repression for some targets even in the absence of the inducer (Cluster 1 in Fig. 2). Finely-graded dCas9 expression may require native, metal-sensitive promoters^53^, or addition of translational-level control such as riboswitches^54^.

The CRISPRi library has a unique advantage over gene knockouts for engineering bioproduction in that the level of essential genes can be titrated, allowing perturbation of the core metabolic network^55^. Cultures where a ‘metabolic switch’ shifts metabolism away from growth can be more productive^14,38,56,57^. By coupling the CRISPRi library to a fluorescence assay, we were able to screen thousands of potential knockdowns for increased productivity, giving a test of many metabolic engineering strategies previously proposed by computational modeling. However, several limitations were apparent in the library-droplet microfluidics workflow. CRISPRi induction must be optimally timed before sorting. The 4 h incubation period where cells secrete L-lactate generates high single-cell variability and is dependent on cell shading, though we attempted to counter this with a high screening depth. This short incubation period was necessary to prevent saturation of the fluorescence assay. Larger droplet sizes would allow for longer incubation times. In batch validation, not all of the enriched clones from the droplet sorting gave higher L-lactate titer and there were apparent tradeoffs between productivity and carbon partitioning in some clones. Therefore, we propose that the CRISPRi library is most useful for revealing principles for guiding engineering, and that clones must be validated.

It is easy to envision expanding an inducible CRISPRi library to other screens. Strains with faster growth rate may not be robust under stress conditions. For example, the *pmgA* and *slr1916* mutants grew faster than the control strain in L100, L300, and LD conditions, but slower under lactate stress. Further, *pmgA* and *slr1916* knockout strains are known to be glucose sensitive in the light, as photosystem stoichiometry cannot adapt to additional reductant supply from sugar metabolism^21,22^. Repression of *ssl2982* increases growth in L300 condition, but a knockout mutant is known to be sensitive to shifts in CO_2_ and temperature^58^. Challenging the CRISPRi library to fluctuating conditions would allow a deeper study of the tradeoff between growth speed and robustness. Since the two fastest-growing mutants found here were also glucose sensitive, it could be worthwhile to add a screen for photomixotrophic growth, which results in flux through alternative glycolytic pathways^59^. Small transposon libraries (300 mutants) have previously been used to identify genes required for phototaxis by screening for colony smearing;^60^ a CRISPRi library could accelerate identification of genes involved in motility. A fluorescence-activated cell sorting (FACS) screen for cell fluorescence could be useful in mapping the photobleaching program during nutrient limitation and screens for recovery ability from starvation could identify carbon metabolism pathways involved in resuscitation^52^. Finally, libraries limited to a subset of target genes would be amenable to multiplexing of gene repression^61^.

## Methods

### Genetic constructs and cloning of sgRNA library

A catalytically dead Cas9 (dCas9, mutations D10A and H840A) from Streptococcus pyogenes was used for gene repression in this study and the *Synechocystis* base strain containing the *tetR*_P_L22__*dCas9* expression cassette in the *psbA1* locus (spectinomycin resistance)^13^. The sgRNA library oligos were synthesized on a 12 K chip by CustomArray Inc., USA (see Supplementary Data 1 for a list of all sgRNA sequences). Plasmid pMD19-T (Takara) was used to create the sgRNA library. First, the homology region around locus *slr0397*, the PL22_sgRNA-NT0 expression cassette, and a kanamycin resistance gene were cloned into pMD19-T by BioBrick assembly. The sgRNA library oligos were then cloned into this vector using Golden Gate assembly. NEB 10-beta Competent *E. coli* cells (New England BioLabs) were used for transformation and ~1,200,000 *E. coli* colonies were obtained. All colonies were collected, resuspended in LB, pooled together, and then cultivated overnight in LB. The plasmid DNA was extracted using the ThermoFisher Maxi plasmid extraction kit. Natural transformation was used to transform the plasmid (10 μg) in *Synechocystis*^62^, where ~300,000 colonies were obtained. Colonies were collected, resuspended in fresh BG-11 and pooled. The pooled *Synechocystis* library was stored at −80 °C. To re-create specific sgRNAs for clone validation, we used overlap-extension PCR of a template sgRNA to introduce the protospacer^13^. All primers are listed in Supplementary Data 7. The L-lactate-secreting *Synechocystis* strain was created by cloning the *ldh* gene with L39R substitution from *Lactococcus lactis* under the P*trc* promoter^32^. This gene construct was inserted into the genome at locus *slr0168* with a chloramphenicol resistance cassette. This strain was subsequently transformed with the *tetR*_P_L22__*dCas9* cassette and sgRNA library as described above, resulting in a L-lactate-producing *Synechocystis* sgRNA library. All strains and plasmids are available from the authors upon request.

### sgRNA library design

Two sgRNAs were designed for each open reading frame (ORF) and non-coding RNA in the *Synechocystis* genome (Reference genome NCBI NC_000911.1, [https://www.ncbi.nlm.nih.gov/nuccore/NC_000911.1]). ORF sequence annotations were obtained from NCBI (downloaded on 18.03.2016). Locations of non-coding RNAs were from Kopf and Hess^15,63^. An in-house Python script was used to create protospacer sequences as close to the transcription start site (TSS) or translation start codon (ATG) as possible, within the following criteria: Target regions were required to be within 500 bp from the known TSS or within 75% of total gene length, absence of G_6_ and T_4_, and GC content between 25 and 75%. Target sequences were searched according to the pattern 5′-CCN[20-25 bases]T-3′. The 5′-CCN ensured a 5′-NGG-3′ PAM site on the coding strand, and 3′ T was to ensure binding of the 5′ end of the sgRNA, known to have an A when transcribed from promoter P_L22_ in *Synechocystis*^64^. For each target fitting these criteria, potential off-target regions in the genome were then identified. An off-target binding site defined as having fewer than two mismatches in the PAM-proximal 17 bp region of the proposed sgRNA. Both NGG and NAG PAMs and both strands were considered. Then all sgRNA candidates for a gene, the two sgRNAs with the least off-targets were selected. If possible, sgRNAs were selected that were at least 10 bp apart from each other. The in-house Python script is publicly available at [https://github.com/KiyanShabestary/2019_CRISPRi_library].

### Cultivation in photobioreactor turbidostats

The *Synechocystis* sgRNA library was cultivated in an 8-tube photobioreactor (Multi-Cultivator MC-1000-OD, Photon System Instruments, Drasov, CZ). The system was customized to perform turbidostat cultivation^16^. Reactors (65 mL) were bubbled with 1% v/v CO_2_ in air (2.5 mL/min) at 30 °C, and light intensity was controlled by a computer program. The OD_720 nm_ and OD_680 nm_ were measured every 15 min. The turbidity set point was OD_720 nm_ = 0.2 and 22 mL fresh BG-11 was added to dilute the culture once the set point was exceeded. Antibiotics (25 µg/mL spectinomycin, 25 µg/mL kanamycin) and anhydrotetracycline inducer (500 ng/mL) was added to the culture and the reserve BG-11 media used for dilution. For LD cultures, the light regime (12 h light − 12 h dark) followed a sinusoidal function with maximum light intensity at 300 µmol m^−2^ s^−1^ (L (t) = 300∗sin (π/43200∗t) where t = cultivation time in seconds). For turbidostat cultivations with added L-lactate, light intensity was 100 µmol m^−2^ s^−1^ and either 100 mM sodium L-lactate or 100 mM NaCl was added to the BG-11 before inoculation and medium pH was adjusted to 7.8. All turbidostat cultivations described in this work were performed as four independent replicates. To sample for NGS, 15 mL of culture volume was harvested by centrifugation (5,000 × *g* for 5 min, 25 °C). Cells were collected and stored at −20 °C. Batch cultivations for growth rate or L-lactate quantification of selected sgRNA mutants were performed in the same conditions as turbidostat cultures, but with light at 300 µmol photons m^−2^ s^−1^. Mutants were pre-cultivated in BG-11 with antibiotics (25 µg/mL spectinomycin, 25 µg/mL kanamycin) and aTc inducer (added to 500 ng/mL) in a climatic chamber (Percival Climatics SE-1100 with 100 μE/s/m^2^ illumination,1% v/v CO_2_ at 30 °C) for 3 days, and then inoculated into the photobioreactor to OD_730 nm_ = 0.05 before growth measurements began.

### Next-generation sequencing of sgRNA region

A two-step PCR procedure was carried out for NGS library preparation using sgRNA library plasmid from *E.coli* or genomic DNA from *Synechocystis* library as template. Genomic DNA was extracted from *Synechocystis* cell pellets (15 mL culture at OD_720 nm_ 0.2) using GeneJET Genomic DNA Purification Kit (Thermo Fisher Scientific). 1st step PCR was performed using the primer pair LUYA593/LUYA594, which amplify sgRNAs from the plasmid or genome and add adaptors for NGS. The PCR product was analyzed on Agilent 2100 Bioanalyzer (Agilent Technologies) and gel purified using GeneJET Gel Extraction Kit (Thermo Fisher Scientific). The purified DNA was used as template to perform the 2nd step PCR using NEBNext Multiplex Oligos for Illumina (Dual Index Primers Set 1) (New England Biolabs), followed by Bioanalyzer analysis and gel purification. Purified DNA was quantified using Qubit fluorometer 2.0 (Thermo Fisher Scientific) and then pooled. The NGS was performed on Illumina NextSeq 500 system using NextSeq 500/550 High Output v2 kit (75 cycles). In a typical NGS run, 40 samples were analyzed simultaneously, providing 50–100 reads per sgRNA per sample (Supplementary Fig. 12). We used sickle 1.33 [https://github.com/najoshi/sickle] to trim (75 nt) and clean reads. A custom python script was used to assign and count reads to each sgRNA, available at [https://github.com/KiyanShabestary/2019_CRISPRi_library].

### Fitness score calculation

The gradual, sgRNA-mediated depletion of clones from the library allows estimation of the contribution to cellular fitness for each individual gene. Here, we defined the fitness *F* of a mutant as the area under the curve (AUC) for log_2_ fold-change sgRNA abundance (log_2_ FC) at a number of generations (n_gen_) since induction, normalized by maximum generations.

1 $$F = \frac{{{\mathrm{AUC}}\left( {{\mathrm{n}}_{{\mathrm{gen}}},{\mathrm{log}}_2{\mathrm{FC}}} \right)}}{{{\mathrm{maximum}}({\mathrm{n}}_{{\mathrm{gen}}})}}$$

2 $$\Delta F = F_{{\mathrm{L}}300} - F_{{\mathrm{L}}100}$$

Differential fitness Δ*F* between two conditions, e.g., L300 and L100, was calculated according to Eqs. 1 and 2.

### RNA extraction and sequencing

*Synechocystis* fast growing mutant strains as well as the control strain (sgRNA-NT0) were cultivated in photobioreactor in turbidostat mode under the same conditions as they were enriched (see above). At 3 days post induction, 40 mL culture (OD_730 nm_ = 0.2) was sampled. Cells were collected by centrifugation for 5 min at 4 °C, and total RNA was extracted immediately afterwards. Ribosomal rRNA was depleted using Illumina Ribo-Zero rRNA Removal Kit (Bacteria). Library preparation was carried out using NEBNext Ultra II Directional RNA Library Prep Kit (New England Biolabs) following manufacturers guidelines. Libraries were sequenced on Illumina NextSeq 500 System using NextSeq 500/550 High Output v2 kit (75 cycles). RNA sequencing reads were filtered and mapped to the genome using Ribopipe^65^. Filtering entailed adapter removal with cutadapt 1.18, quality trimming with sickle 1.33, and rRNA and tRNA removal using Bowtie 1.2.2 and reference sequences from the *Synechocystis* genome (Reference genome NCBI NC_000911.1, [https://www.ncbi.nlm.nih.gov/nuccore/NC_000911.1]). Bowtie v. 1.2.2 was used to map the filtered reads to the *Synechocystis* genome. The number of reads mapping to each gene in each sample was counted and formatted using custom Python and R scripts available at [https://github.com/Asplund-Samuelsson/ribopipe]. RNA sequencing data were initially processed as described before for NGS sequencing of library data. DESeq2 was used to determine fold changes between conditions as well as significance (three independent biological replicates). Significant genes were selected based on the following two criteria: absolute log_2_ FC ≥ 1, adjusted *p*-value ≤ 0.05). Unsupervised clustering of significantly different genes based on expression in all mutants was performed as described for NGS data analysis.

### Statistical analysis of library competition data

All analyses were performed using the R programming language and are documented in R markdown notebooks available at [https://m-jahn.github.io/]. Data for all competition experiments performed with the sgRNA library can be accessed at [https://m-jahn.shinyapps.io/ShinyLib/]. First, data tables from different sequencing runs were merged into a single master table. For simplicity, harvesting time-points 12 and 30 days for the sodium chloride condition (NACL) were re-labelled as 16 and 32 days to correspond to time-points of all other samples. This did not influence the calculation of generation time or fitness score, and was done only to display these samples along with corresponding L-lactate samples. The R package DESeq2 was used to determine fold changes between conditions as well as significance metrics (multiple hypothesis adjusted *p*-value, Benjamini-Hochberg procedure)^66^. Fold changes and *p*-values were determined using four independent biological replicates for all cultivations. Gene-wise annotation was added based on Uniprot (IDs, protein properties, GO terms) and CyanoBase (functional categories). Altogether 7119 unique sgRNAs corresponding to 3541 unique genes (without non-coding RNAs) were included in the analysis. The coverage per sample in terms of quantified sgRNAs and median read count per gene is shown in Supplementary Fig. 12.

### Unsupervised clustering of sgRNA data

To cluster sgRNAs based on depletion/enrichment pattern, a dissimilarity matrix was computed using R’s dist function with distance measure euclidean. Clustering was performed using function hclust with method ward.D2. Silhouette analysis was performed to find the optimal number of clusters (silhouetteAnalysis from package silhouette) and showed equally good separation for 3–9 clusters. A number of six clusters was chosen representing sgRNAs with decreasing level of depletion (1–4) as well as two clusters with unchanged sgRNAs (5 and 6). These two clusters were separated by a group of sgRNAs (around 25% of all) that showed a spurious, temporary enrichment for two specific time-points (1 and 2 day measurements). This temporary enrichment was most likely due to technical variation (e.g., in sample preparation) and has no biological explanation. Clusters 5 and 6 were therefore combined in cluster 5 (Fig. 2a).

### Gene-ontology enrichment

For gene-ontology (GO) term enrichment, the TopGO package by Alexa et al. was used to determine GO terms associated with sgRNAs for clusters 1–4 (TopGO method ‘Fisher, eliminating’)^67^. The resulting list of GO terms was filtered by dispensability scores obtained using REVIGO (http://revigo.irb.hr/, threshold ≤ 0.5). Furthermore, GO terms annotated with less than 5 or more than 200 unique sgRNA/genes or *p*-value > 0.03 were filtered out.

### Enrichment of sgRNA mutants with increased L-lactate tolerance

To find genes involved in L-lactate tolerance, sgRNAs enriched specifically for presence of sodium L-lactate but not sodium chloride were selected (threshold: log_2_ FC ≥ 2, −log_10_ *p*-value ≥ 20). Twenty-four and 75 sgRNAs were found to be enriched after 16 and 32 days, respectively (Fig. 5a). The 24 sgRNAs enriched after 16 days were included in the set of 75 sgRNAs enriched after 32 days. In contrast to the 75 sgRNAs that were enriched, thousands of sgRNAs were depleted during growth in the presence of L-lactate. To find sgRNAs specifically depleted under L-lactate but not sodium chloride, a differential fitness score was calculated between the two conditions (*dF* = *F*_*NaCl*_ *−* *F*_*Lac*_). The top 200 sgRNAs with highest *dF* were selected and six genes were found with both sgRNAs strongly depleted (see also Supplementary Fig. 9).

### Estimation of mutant growth rates

The change in a mutant’s abundance within the total population, combined with the population average growth rate (known from the dilution rate of the turbidostat), allows estimation of the growth rate of each mutant. The rate of enrichment or depletion of a mutant (*μ*_*diff*_) was defined as the population growth rate (*μ*_*pop*_) subtracted by the mutant’s individual growth rate (*μ*_*mut*_) (Eq. 3).

3 $${\mathrm{\mu }}_{{{diff}}} = {\mathrm{\mu }}_{{{pop}}} - {\mathrm{\mu }}_{{{mut}}}$$

The depletion of a mutant from the library can be modeled as a function of time, where the mutant fraction *f* at time point *t* becomes:

4 $$f(t) = f(t = 0) \times \left( {1 - ({\mathrm{\mu }}_{{{pop}}} - {\mathrm{\mu }}_{{{mut}}})} \right)^t$$

The mutant fraction *f* at different times and the average growth rate of the population are known parameters, allowing to estimate growth rate for all mutants (Eq. 4).

### Correlation between gene expression variability and fitness

To correlate gene fitness (obtained from sgRNA library competition experiments) with variability in gene expression, the proteomics dataset PRIDE PXD009582 was used^16^. The dataset contains mass spectrometry based protein measurements of *Synechocystis* for 2000 proteins at five different growth rates, each for CO_2_ and light limitation. The variability of a protein was defined as growth-rate dependent change in abundance, and determined as *p*-value from analysis of variance (ANOVA). If a protein changed abundance significantly with growth rate (either up or downregulation) a lower *p*-value was obtained. Proteins were binned into groups according to *p*-value ranges (1–0.1, 0.1–0.05, 0.05–0.01, 0.01–0.001, <0.001) and fitness score of sgRNAs associated with the respective proteins in a group were plotted.

### Absorption spectrum of L-lactate-tolerant mutants

Selected mutants as well as the control strain (sgRNA-NT0) were cultivated in 30 mL BG-11 supplemented with 100 mM sodium L-lactate in shaking flasks at 100 µmol photons m^−2^ s^−1^ continuous light, and 500 ng/mL aTc was added at the beginning of the cultivation. 0.5 mL cell culture was sampled after induction for 3 days and cells were collected by centrifugation at 5000 × *g* for 10 min. Cells were then washed twice with 1 mL PBS by centrifugation and resuspension, and then resuspended in 400 µL PBS. Two times 100 µL resuspended cells were transferred to a transparent 96-well plate. Absorption spectra of cell samples were obtained in the range of 350–800 nm using a photospectrometer (SpectraMax M5, Molecular Devices). Absorption spectra were normalized to the reference A_720 nm_ and relative chlorophyll a, phycocyanin and carotenoid content of cells were determined using the ratio of A_680 nm_/A_720 nm_, A_630 nm_/A_720 nm_, and A_490 nm_/A_720 nm_, respectively.

### Droplet microfluidic screening of L-lactate-producing sgRNA library

The L-lactate-producing *Synechocystis* sgRNA library was grown in shake-flasks in a climatic chamber. Cultures were supplemented with antibiotics (12.5 µg/mL chloramphenicol, 25 µg/mL spectinomycin) and aTc (1 μg/mL). For the productivity assay, cells were harvested (OD_720 nm_ = 0.4-0.6), washed to OD_720 nm_ = 0.15, and encapsulated into droplets^68^. A mix of HFE-7500 oil and 1% (w/w) EA surfactant droplet stabilizer (RainDance Technologies) was loaded into a Gastight 5-mL glass syringe (Hamilton). Cells were encapsulated in 10 pL droplets using a flow rate of 400 μL/h for the aqueous solution and 2000 μL/h for the oil. The emulsion was collected in a 1-mL plastic syringe at a withdrawal flow rate of 2000 μL/h. The syringes were connected to the chip by polyether ether ketone tubing, and flow rates were controlled by neMESYS syringe pumps (Cetoni GmbH). The droplet emulsion was incubated in an illuminated syringe (approximately 150 μE/s/m^2^) for 4 h for L-lactate secretion. Three pL of a fluorescence-based L-lactate assay mixture (Cayman Chemical) was then injected into each droplet using a picoinjection chip^69^. The emulsion was injected at a flow rate of 70 μL/h, spacer oil separated the droplets at a flow rate of 500 μL/h and the lactate assay mixture was injected at 30 μL/h. The picoinjected emulsion was collected in a 1 mL plastic syringe protected from light. After 40 min of picoinjection, the collected emulsion was gently mixed and re-injected into a droplet sorting device(Supplementary Fig. 9). The flow rates used were 1000 µL/h for the spacer oil and side oil, and the emulsion was injected at 100 µL/h, corresponding to a droplet sorting rate of 1.5 kHz. Sorted droplets were collected using a withdrawal rate of 1000 µL/h. Approximately 1,800,000 droplets were screened and the top 2% most fluorescent droplets were sorted, corresponding to approximately 36,000 droplets. After sorting, the collected emulsion was broken with 10% v/v 1 H, 1 H, 2 H, 2H-perfluoro-1-octanol (Sigma Aldrich), followed by centrifugation and evaporation to remove the residual liquid. Cells were resuspended in DMSO and heated at 95 °C for 2 min to release genomic content. The cell lysate was used as template to directly amplify the sgRNA region using primer pair LUYA271/LUYA300. Then adaptors were incorporated into the PCR product using primer pair LUYA593/LUYA594, followed by NGS library preparation as described above.

### Droplet microfluidics NGS data filtering

Using NGS obtained on each sorted droplet fraction, we could map 5 million reads per sample, covering to 8000–10,000 unique sgRNAs. sgRNAs with fewer than 32 mapped reads in a sample were removed from that sample. To calculate ‘enrichment factors’ for each sgRNA, the relative abundance of the sgRNA in the sorted droplet was divided by its relative abundance in the total library before droplet encapsulation. Next, sgRNAs where the enrichment factor differed ≥10-fold between replicates were removed. The remaining sgRNAs were then ranked by enrichment factor for each sorted sample. sgRNAs at least three times more abundant in sorted fraction relative to unsorted fraction were given a score of 1 for that sample. Clones with a score 2/4, summed over all samples were determined to have potential to increase L-lactate productivity.

### L-lactate production in batch and photonfluxostat

The L-lactate-producing *Synechocystis* sgRNA library mutants were first cultivated in shake-flasks in a climatic chamber. Cultures were pre-cultivated in BG-11 supplemented with antibiotics (12.5 µg/mL chloramphenicol, 25 µg/mL kanamycin) and induced with aTc (1 μg/mL) 2 days prior to the start of the experiment. Then mutants were transferred to shake-flasks or in an 8-tube photobioreactor (see library cultivation methods) with a starting OD_720 nm_ of 0.1, supplemented with aTc and antibiotics as stated above. Cultures in the photobioreactor were grown in a ‘photonfluxostat’ mode, where light is increased based on cellular density to extend log phase. The amount of light given to the culture is equal to the cellular density multiplied by a light regime factor (1000 µmol photons m^−2^ s^−1^ OD_720 nm_^−1^). L-lactate was measured using a L-lactate fluorescent kit (Cayman Chemical) according to the manufacturer’s instructions.

### Reporting summary

Further information on research design is available in the Nature Research Reporting Summary linked to this article.

## Supplementary information

Supplementary Information

Peer Review

Reporting Summary

Description of Additional Supplementary Files

Supplementary Data 1

Supplementary Data 2

Supplementary Data 3

Supplementary Data 4

Supplementary Data 5

Supplementary Data 6

Supplementary Data 7

## Data Availability

A reporting summary for this Article is available as a Supplementary Information file. Data supporting the findings of this work are available within the paper and its Supplementary Information files. The datasets generated and analyzed during the current study are available from the corresponding author upon request. The data for competition experiments performed with the sgRNA library can also be accessed through an interactive web application [https://m-jahn.shinyapps.io/ShinyLib/]. All sequencing data, including the RNA sequencing data described in Fig. 4, are available at the European Nucleotide Archive with accession number PRJEB35238. Proteomics data used in Supplementary Fig. 1 was from PRIDE PXD009582. The source data underlying Figs. 2, 3, 4b–d, 5a, b, and 6, Table 1, as well as Supplementary Figs 1–7, 9, and 11 are provided as a Source Data file.

## Source data

Source Data

## Code availability

All analyses of sgRNA library competition data were performed using the R programming language and are documented in R markdown notebooks [https://m-jahn.github.io/]. The code used to process RNA-Seq data from raw files (read trimming, mapping to genome, PCA analysis) is deposited at GitHub [https://github.com/Asplund-Samuelsson/ribopipe]. The code used to design sgRNA sequences for each gene in *Synechocystis*, the script to assign NGS reads to sgRNAs, and the pipeline for data filtering doplet NGS data is also available at GitHub [https://github.com/KiyanShabestary/2019_CRISPRi_library].
