# Supplementary material for: Pooled CRISPRi screening of the cyanobacterium Synechocystis sp PCC 6803 for enhanced industrial phenotypes
Source: Nat Commun. 2020 Apr 3;11:1666. doi: 10.1038/s41467-020-15491-7 (PMC7125299; doi:10.1038/s41467-020-15491-7)
Supplement: Supplementary file 1 — Supplementary Information [file 41467_2020_15491_MOESM1_ESM.pdf]

Pooled CRISPRi screening of the cyanobacterium *Synechocystis* PCC 6803 for enhanced industrial phenotypes

Yao *et al.*

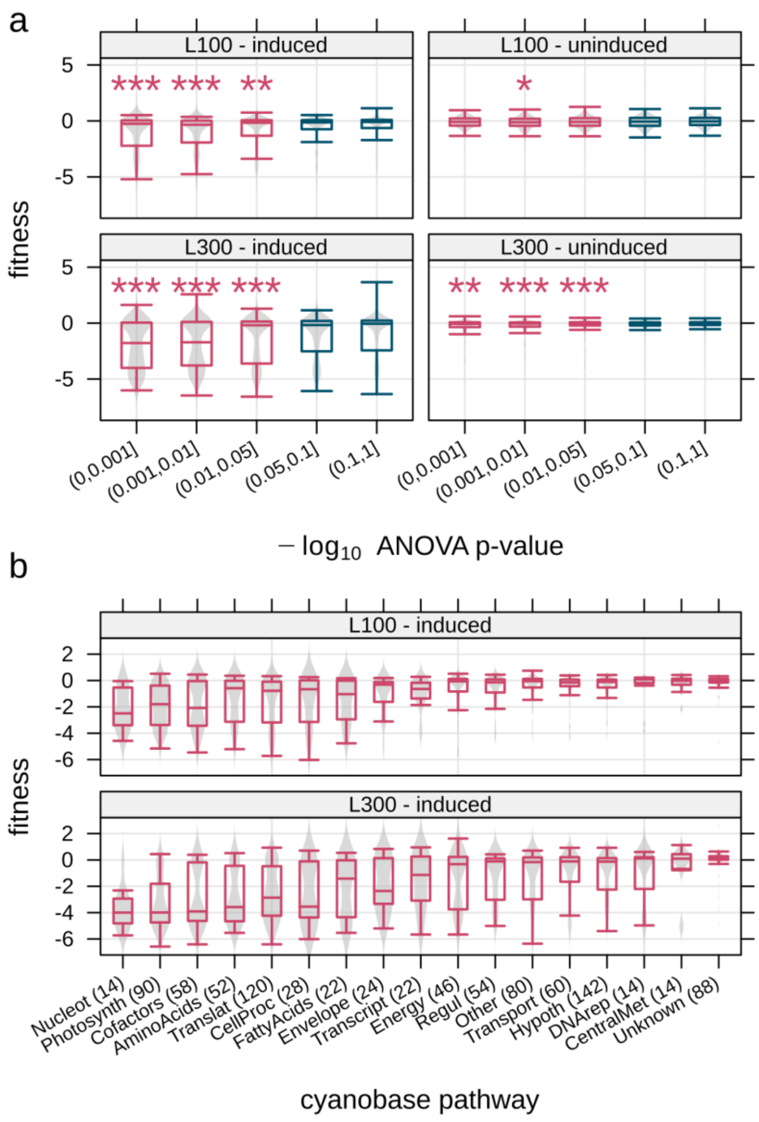

Supplementary Figure 1. Highly regulated proteins have lower average fitness. a Fitness score of sgRNAs broken down by protein variability. Protein variability was estimated using a proteomics study with measurement of protein abundance over several light conditions [1]. Here, the adjusted p-value from ANOVA over all light conditions was used as a metric for variability. For this comparison every sgRNA was mapped to its corresponding protein. n ≥ 208 independent sgRNAs. Red, proteins with ANOVA *p-value* ≤ 0.05. Blue, proteins with ANOVA *p-value* > 0.05. Symbols, significance of distribution being different from last group (0.1-1) according to Student's two-sided t-test: '*' *p-value* ≤ 0.05, '**' *p-value* ≤ 0.01, '***' *p-value* ≤ 0.001 **b** Fitness score of sgRNAs associated with most significantly changing proteins (*p-value* ≤ 0.05, red in **a**), broken down by cyanobase pathways. In brackets, number of unique sgRNAs per pathway (n ≥ 14 independent sgRNAs). Box-and-whisker plots represent the median (horizontal line), range between first and third quartile (box), and minimum and maximum (whiskers) of the fitness score distribution. Source data are provided as a Source Data file.

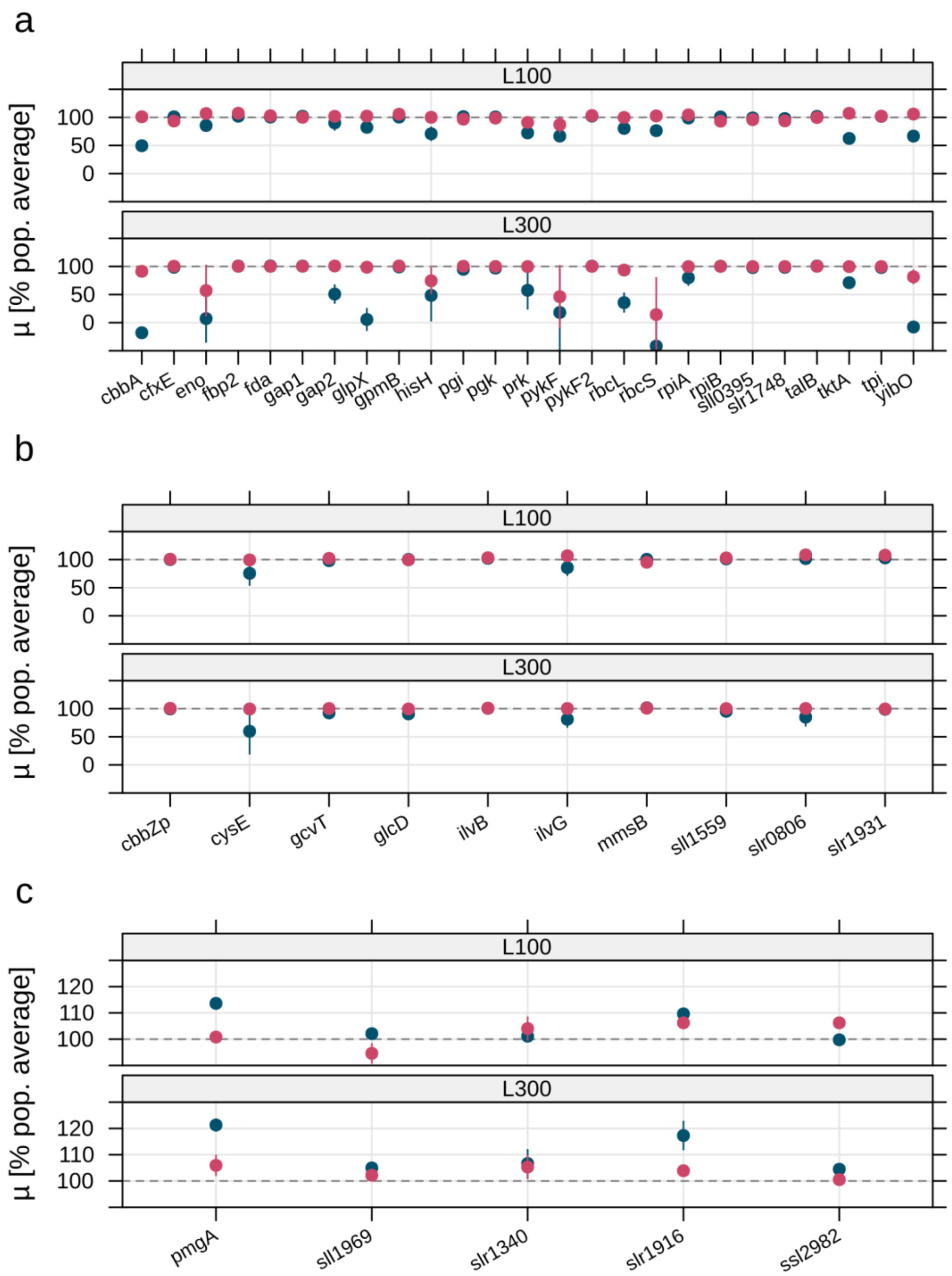

Supplementary Figure 2. Estimated growth rate of selected mutants. Mutant growth rate was determined from depletion of sgRNAs over time as described in Methods (mean and standard deviation of two sgRNAs per gene, each calculated from n=4 replicate cultivations). Growth rate is displayed as % of population growth rate. Red - uninduced control, blue - induced culture. **a** Selected genes from the Calvin cycle. **b** Selected genes for photorespiration. **c** Selected faster growing mutants. Error bars are standard deviations. Source data are provided as a Source Data file.

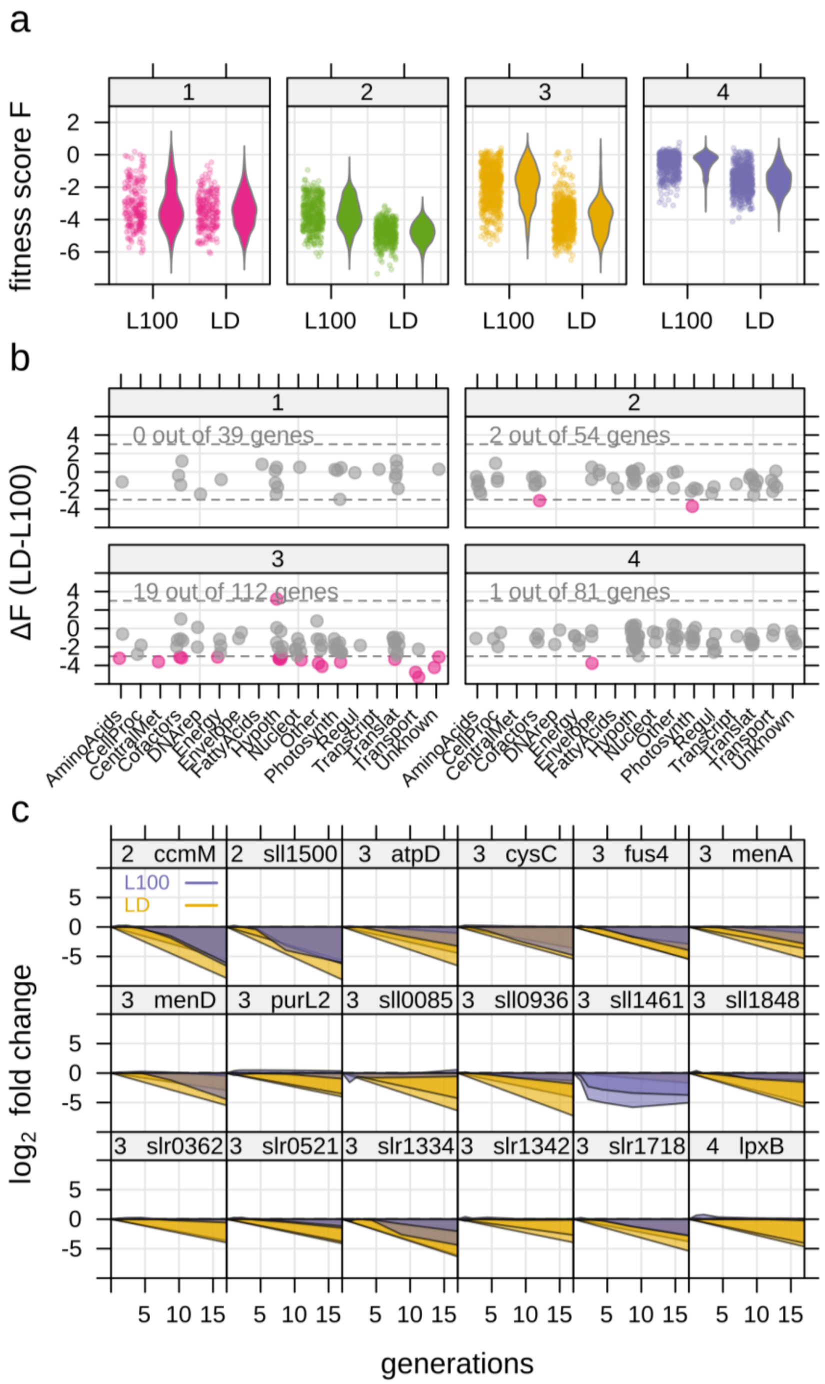

Supplementary Figure 3. Genes with condition-dependent fitness for L100 and LD. a Distribution of fitness score *F* for all sgRNAs in cluster 1 to 4. Fitness score indicates the degree of enrichment (positive) or depletion (negative) of an sgRNA. Comparison between low light (L100 - light with 100 µmol photons m^{-2} s^{-1}) and light-dark cycle (LD - light with 0-300 µmol photons m^{-2} s^{-1}). **b** Difference between fitness score of L100 and LD (Δ*F*) for genes with both sgRNAs in the same cluster. Differentially depleted/enriched sgRNAs indicated in red, threshold: 3 ≤ ΔF ≤ -3. **c** Selection of 18 genes with Δ*F* above threshold as described in **b**. Only genes with 2 depleted/enriched sgRNAs were selected. Source data are provided as a Source Data file.

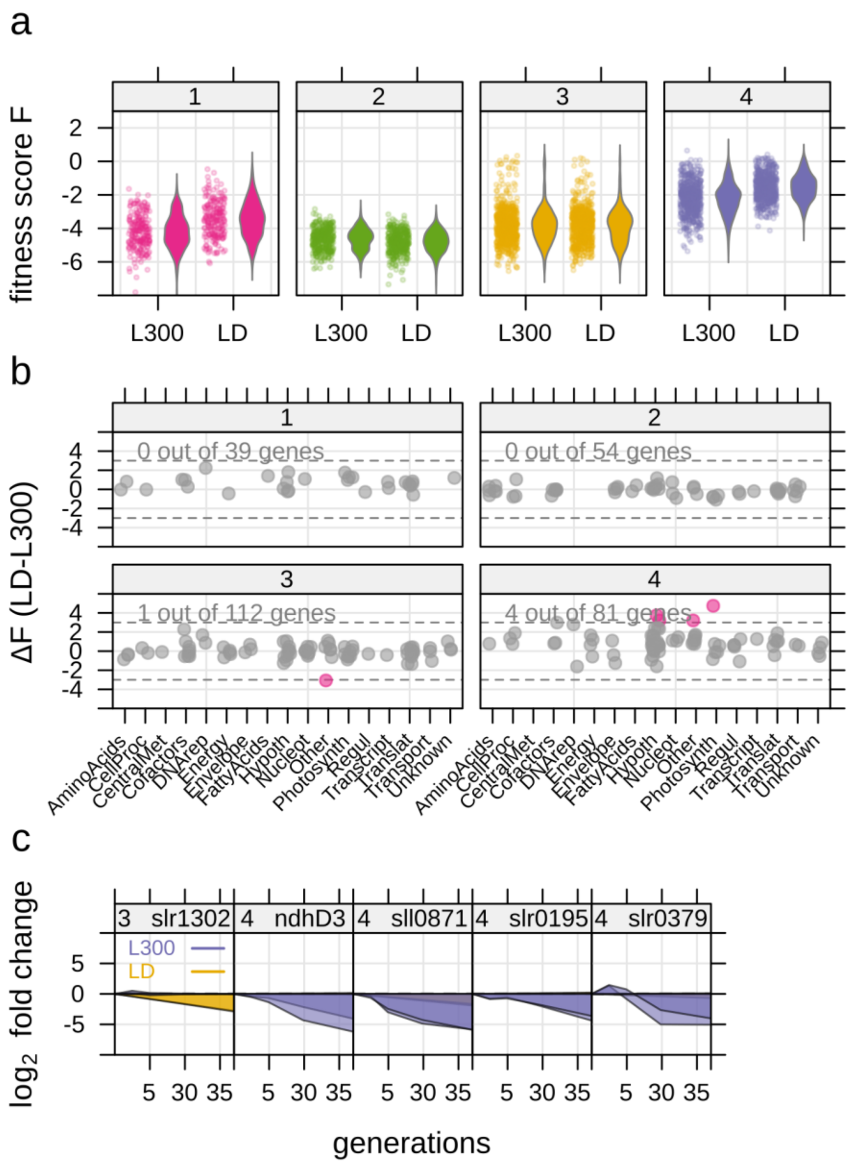

Supplementary Figure 4. Genes with condition-dependent fitness for L300 and LD. a Distribution of fitness score *F* for all sgRNAs in cluster 1 to 4. Fitness score indicates the degree of enrichment (positive) or depletion (negative) of an sgRNA. Comparison between high light (L300 - light with 300 µmol photons m^{-2} s^{-1}) and light dark cycle (LD - light with 0-300 µmol photons m^{-2} s^{-1}). **b** Difference between fitness score of L300 and LD (Δ*F*) for genes with both sgRNAs in the same cluster. Differentially depleted/enriched sgRNAs indicated in red, threshold: 3 ≤ ΔF ≤ -3. **c** All genes with Δ*F* above threshold as described in **b**. Only genes with 2 depleted/enriched sgRNAs were selected. Source data are provided as a Source Data file.

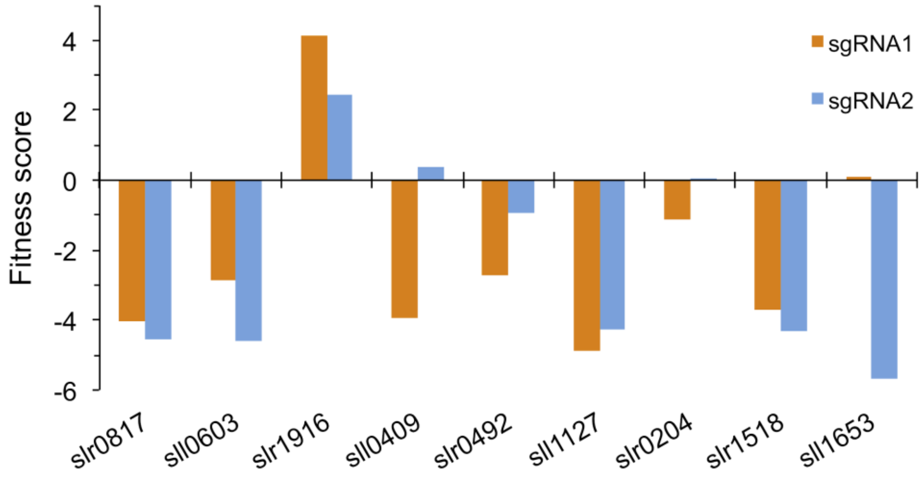

Supplementary Figure 5. Fitness scores of sgRNAs targeting phylloquinone biosynthetic pathway genes. *slr0817*: isochorismate synthase (MenF); *sll0603*: SEPHCHC synthase (MenD); *slr1916*: (*1R,6R*)-2- succinyl-6-hydroxy-2,4-cyclohexadiene-1-carboxylic acid synthase (MenH); *sll0409*: *o*-succinylbenzoate synthase (MenC); *slr0492*: *o*- succinylbenzoate-CoA ligase (MenE); *sll1127*: 1,4-dihydroxy-2-naphthoate synthase (MenB); *slr0204*: 1,4-dihydroxy-2-naphthoate-CoA thioesterease; *slr1518*: 1,4-dihydroxy- 2-naphthoate prenyltransferase (MenA); *sll1653*: demethyl-menaquinone/demethyl-phylloquinone methyl- transferase (MenG). Source data are provided as a Source Data file.

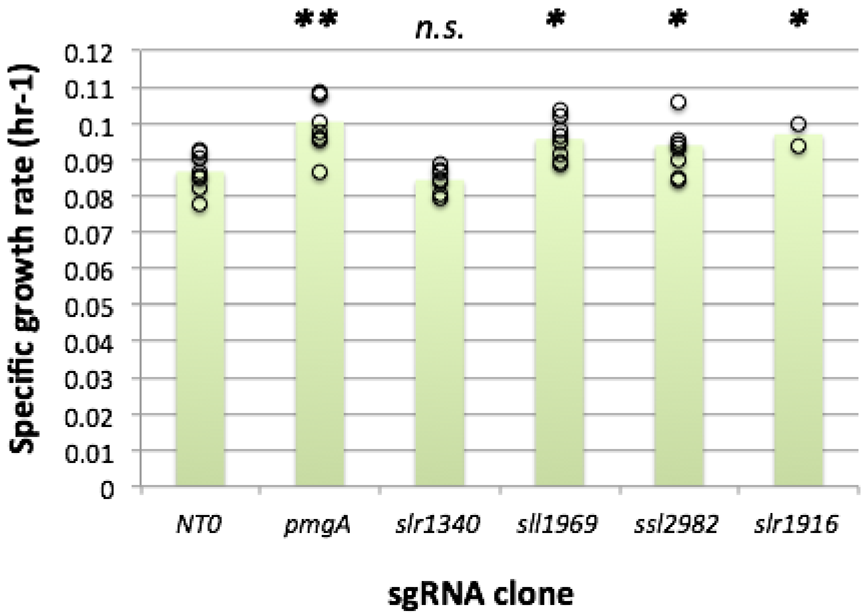

§

Supplementary Figure 6. Calculated maximum growth rates for selected sgRNA clones, compared to a control strain. NT0: control strain with nonsense sgRNA. CRISPRi clones were reconstructed and grown in batch culture, except for *slr1916* which was cultivated in turbidostat (OD_{730} = 0.1). CRISPRi was induced 2 days before inoculation. Cells were inoculated to OD_{730} = 0.05. Culture conditions in each case were 30°C, 300 µmol photons m^{-2} s^{-1} illumination, and 1% CO_2 in MC-1000-OD bioreactors (Photon Systems Instruments, Czech Republic). The OD_{720} was recorded every 15 minutes, and growth rate was estimated from the slope of the log transformed OD_{720} data during exponential phase. For strains *NT0, pmgA, slr1340, sll1969,* and *ssl2982*, two colonies were picked from the transformation plate. Then, four separate batch cultivations were started from each clone in the MC-1000 OD clones (8 biological replicates). Technical failure in one set of cultivations resulted in n=7 biological replicates used for growth rate calculation. For *slr1916,* one colony was used to inoculate two turbidostats (n=2 biological replicates). Bars are the mean value of calculated growth rate, with individual calculated growth rates shown as points. P-values for growth rates were calculated by comparing to growth rate of NT0, using Students t-test (unpaired), two-sided. '*' *p-value* ≤ 0.05, '**' *p-value* ≤ 0.01. N.s. not significant. Source data are provided as a Source Data file.

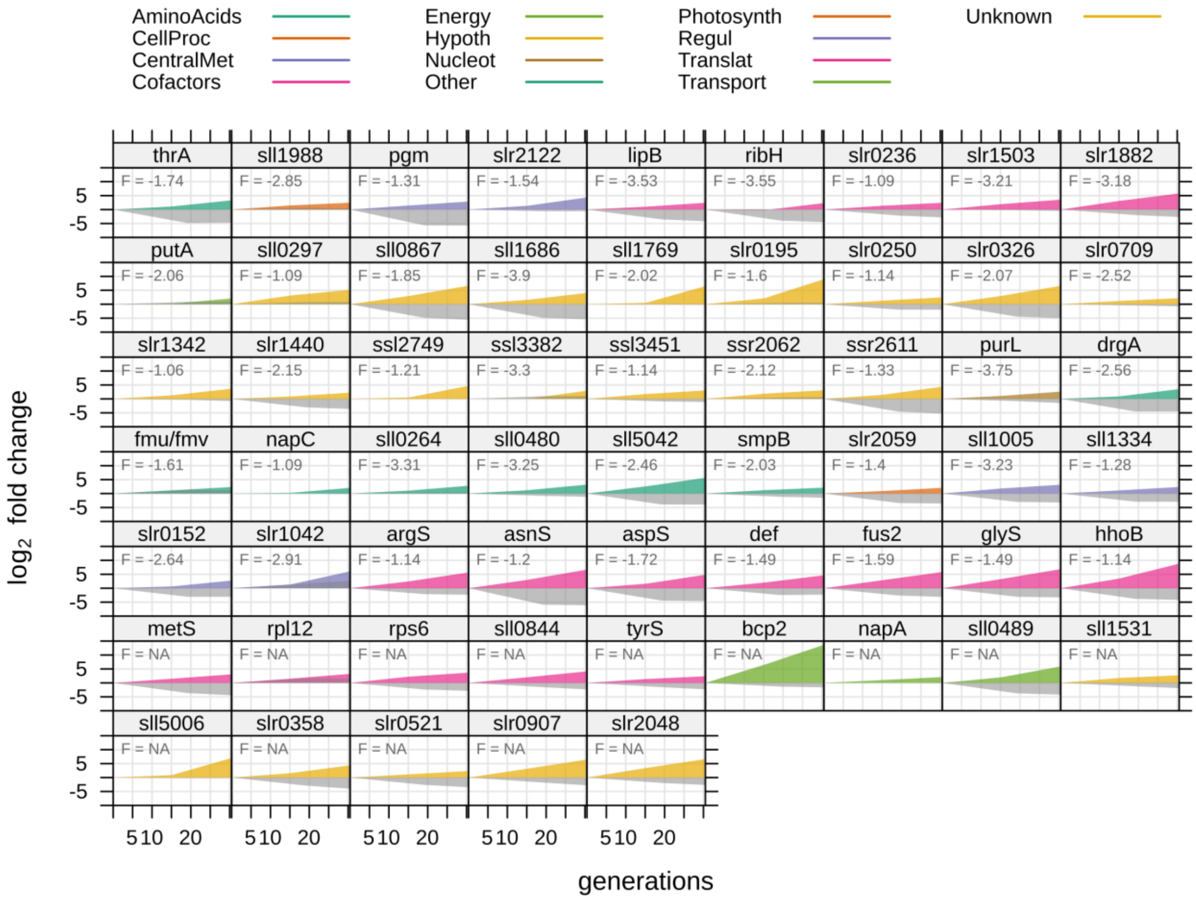

Supplementary Figure 7. Genes with only 1 sgRNA enriched when L-lactate was present in culture medium. Depicted is the $\log_2$ fold change over number of generations the cells were cultivated in a turbidostat bioreactor. Colored area: sgRNA in presence of 0.1 M L-lactate, color-coded by cyanobase functional category, grey area: sgRNA in presence of 0.1 M sodium chloride. *F*, fitness score for sgRNA in presence of lactate. Fitness score is positive for enrichment and negative for depletion of sgRNAs. Source data are provided as a Source Data file.

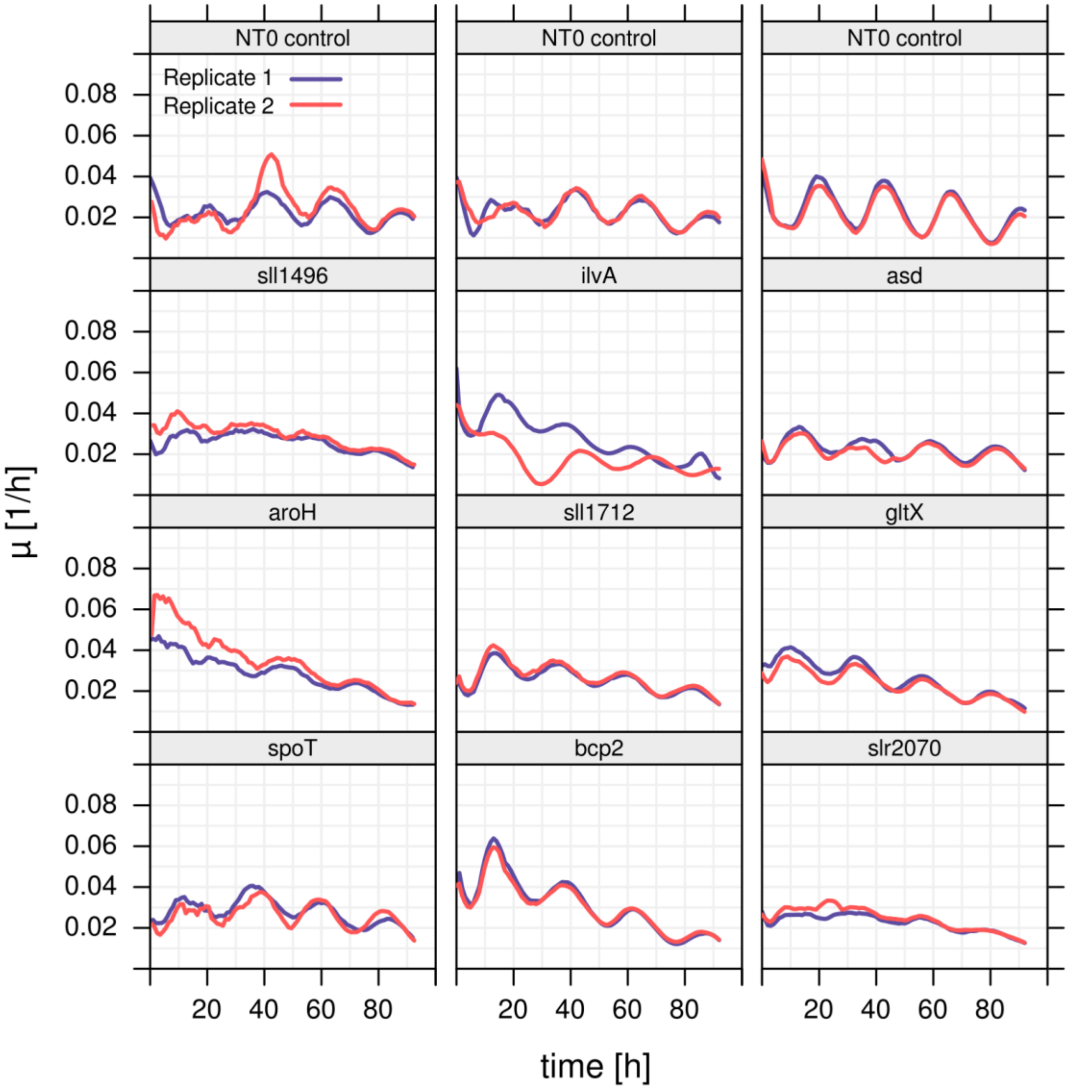

Supplementary Figure 8. Cultivation of L-lactate tolerant sgRNA mutants for initial 100 hours. Selected sgRNA mutants were reconstructed and grown in axenic turbidostat cultivations in presence of 0.1 M L-lactate (n = 2). Cultivation conditions for all strains were 100 µmol photons m^{-2} s^{-1} and 1% CO_2. The control strain (NT0) contains an sgRNA with no target site in *Synechocystis* genome. Specific growth rate µ over time was calculated in a sliding window of step width 5 hours. The oscillation in growth rate is caused by the inherent circadian rhythm.

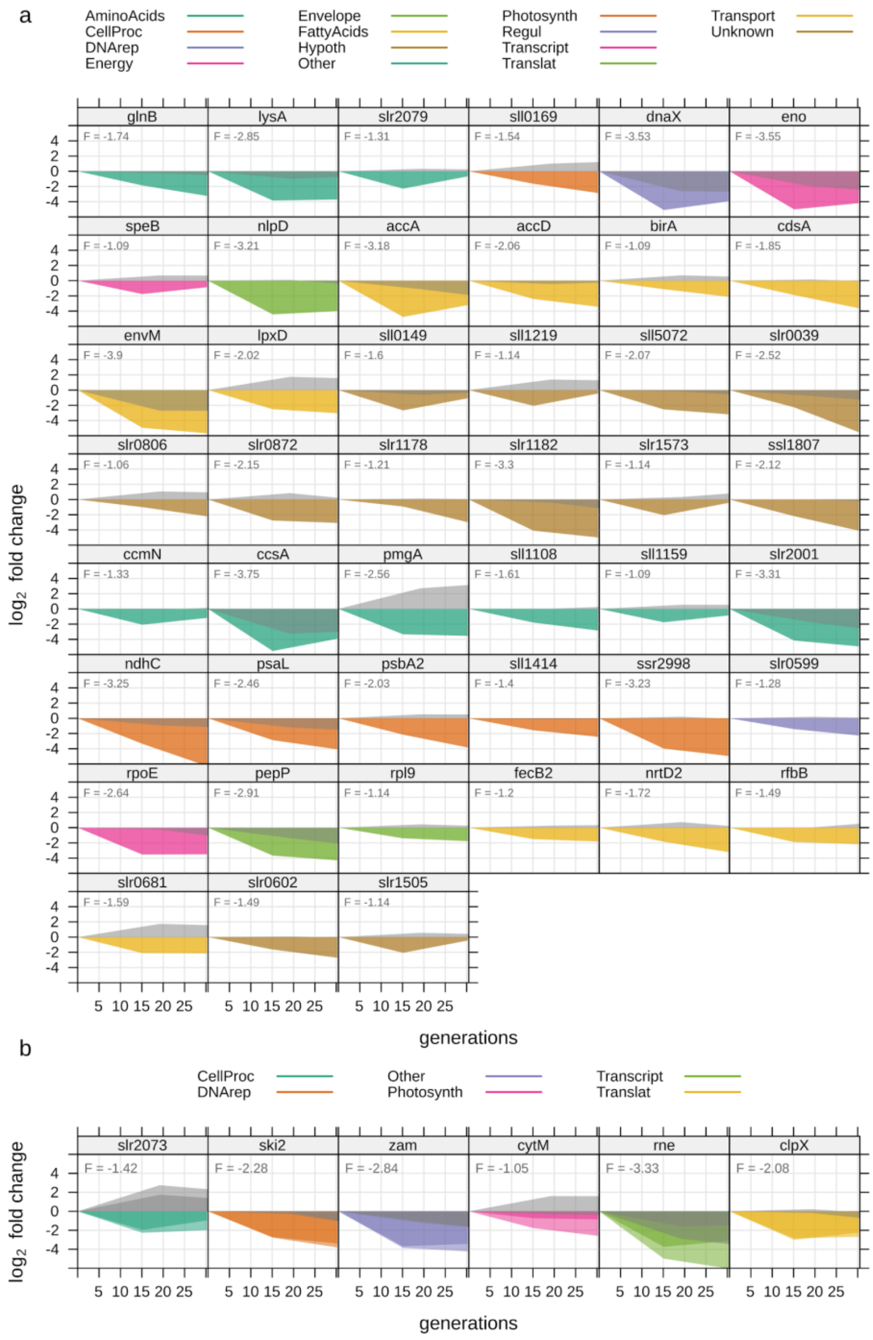

Supplementary Figure 9. Genes that were depleted when L-lactate was present in culture medium. **a** Genes where only 1 sgRNA was depleted. **b** Genes where both sgRNAs were depleted. Depicted is the $\log_2$ fold change over number of generations when the cells were cultivated in a turbidostat bioreactor. Colored area: sgRNA in presence of 0.1 M L-lactate, color-coded by cyanobase functional category, grey area: sgRNA in presence of 0.1 M sodium chloride. *F*, fitness score for sgRNA in presence of L-lactate. Fitness score is positive for enrichment and negative for depletion of sgRNAs. Source data are provided as a Source Data file.

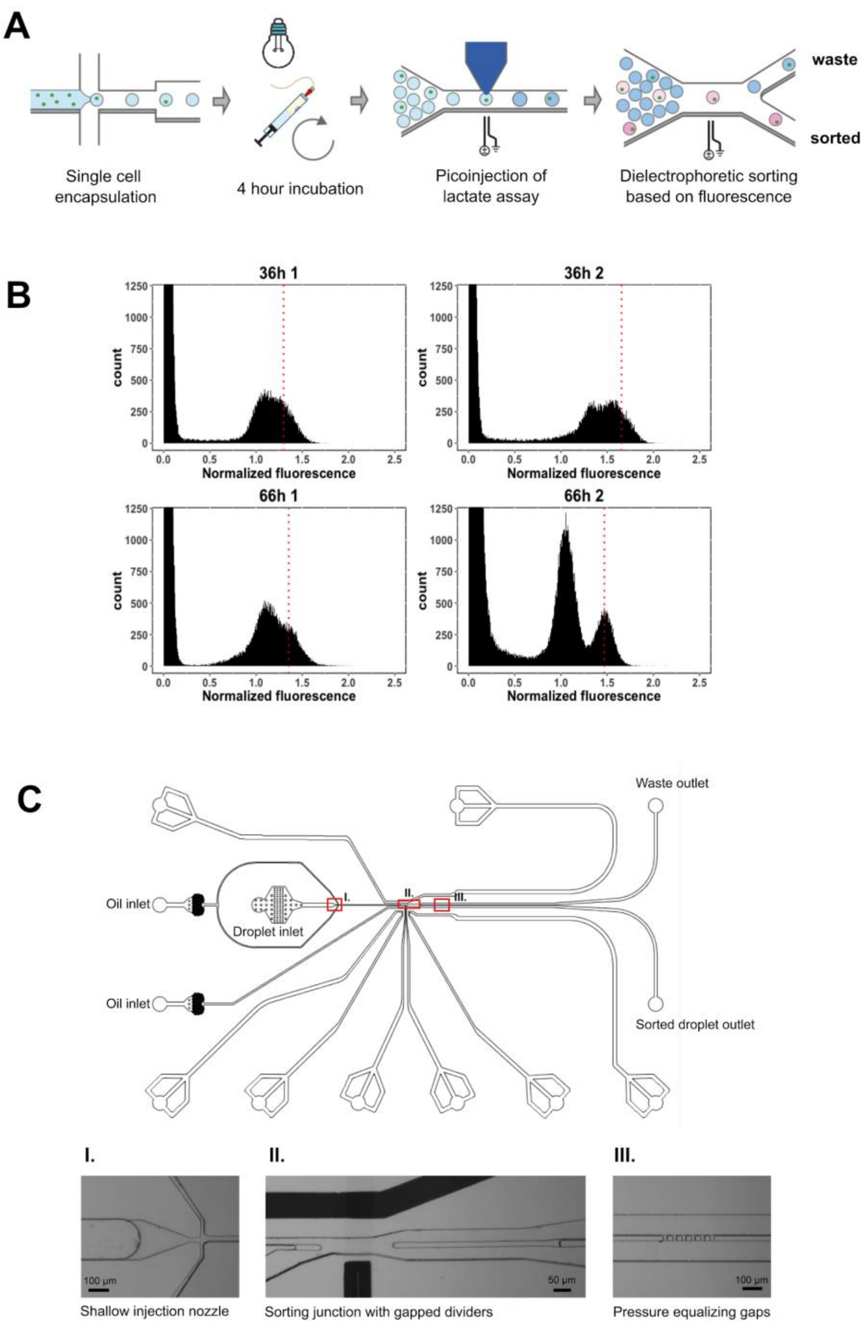

Supplementary Figure 10. Method details on droplet sorting. a Schematic of microfluidics-based sorting of lactate producers library. **b** Histogram of lactate fluorescence intensity from droplets at different time points after dCas9 induction. Dotted line - threshold between low (left) and high (right, sorted droplets) fluorescent sub-populations. **c** Diagram of microfluidic droplet sorter.

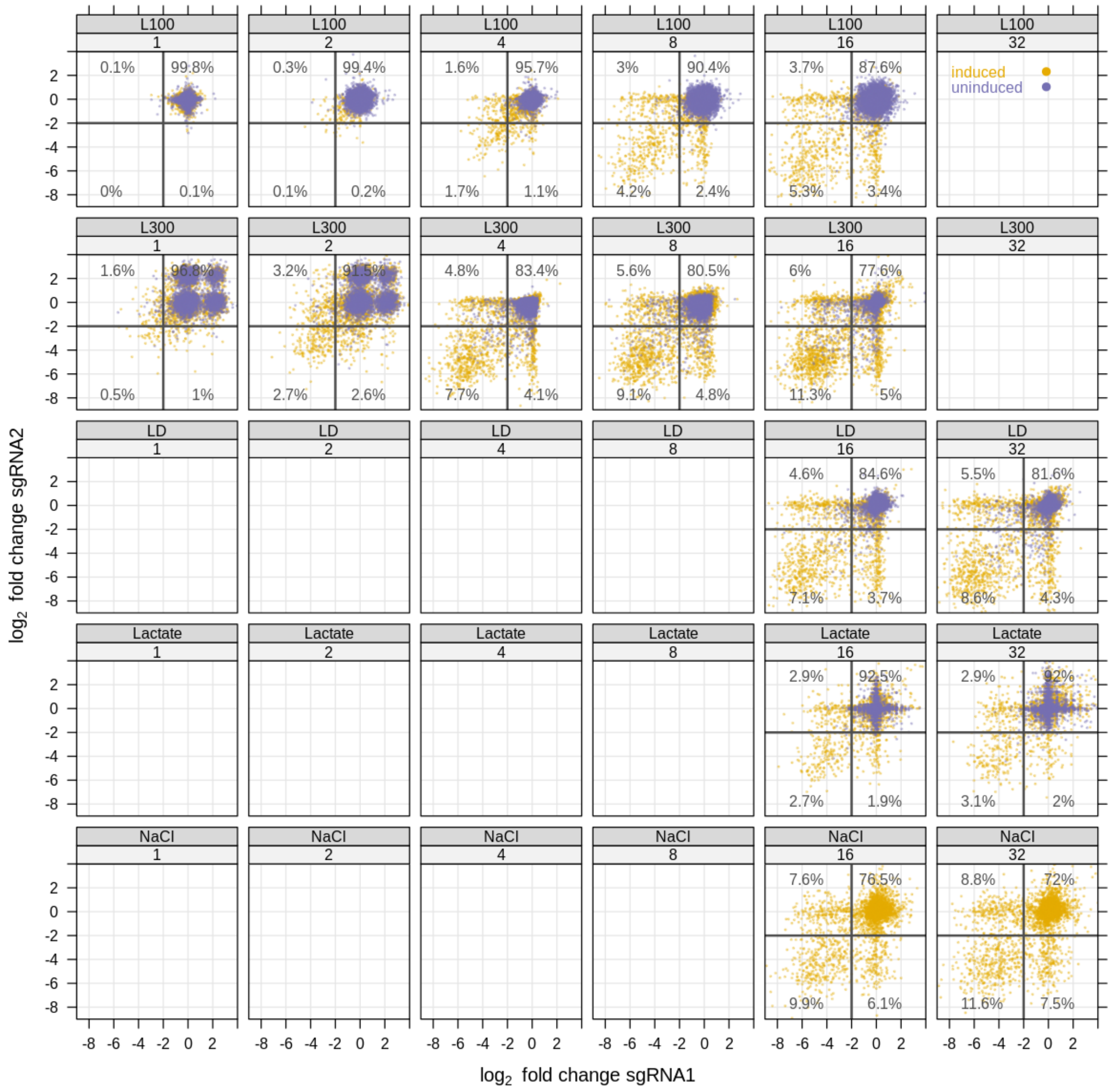

Supplementary Figure 11. Correlation of sgRNA pairs for each gene. Correlation of the $\log_2$ fold change of sgRNA1 *versus* sgRNA2 for each gene, broken down by condition. Panels show an overlay of induced (blue) and uninduced (red) samples. Panel numbers indicate time in days. L100 - light with 100 μmol photons m^{-2} s^{-1}, L300 - light with 100 μmol photons m^{-2} s^{-1}, LD - light-dark cycle, Lactate - addition of 0.1 M L-lactate, NaCl - addition of 0.1 M sodium chloride. Source data are provided as a Source Data file.

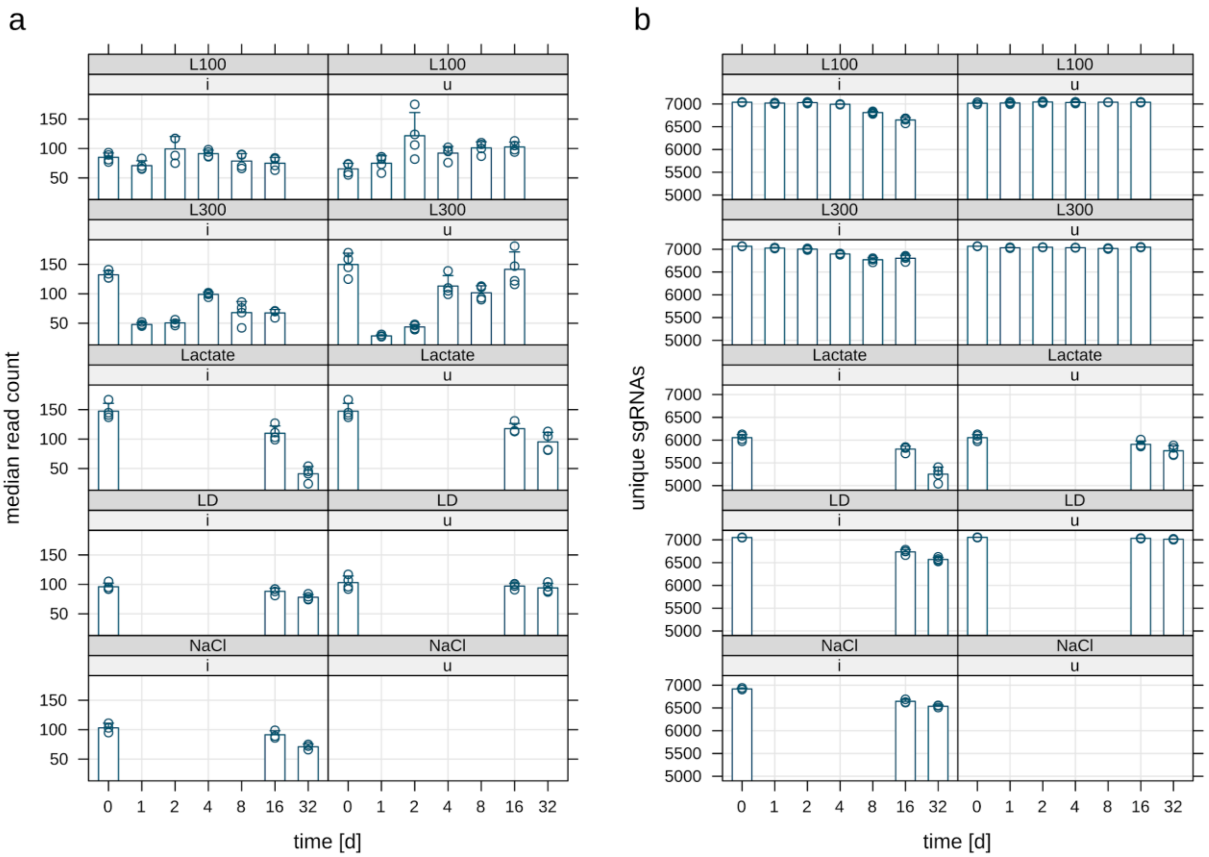

Supplementary Figure 12. Median read count and coverage of samples in terms of quantified unique sgRNAs for each condition and time point. a Median read count per condition. Bars and whiskers correspond to mean and standard deviation of 4 biological replicates. **b** Coverage of samples in terms of quantified unique sgRNAs per condition and time point. Bars and whiskers correspond to mean and standard deviation of 4 biological replicates. L100 - light with 100 µmol photons m^{-2} s^{-1}, L300 - light with 300 µmol photons m^{-2} s^{-1}, LD - light-dark cycle, Lactate - addition of L-lactate, NaCl - addition of sodium chloride.

Supplementary References

1. Jahn M*, et al.* Growth of cyanobacteria is constrained by the abundance of light and carbon assimilation proteins. *Cell Rep* **25**, 478-486 e478 (2018).
