# Supplementary material for: Pooled CRISPRi screening of the cyanobacterium Synechocystis sp PCC 6803 for enhanced industrial phenotypes
Source: Nat Commun. 2020 Apr 3;11:1666. doi: 10.1038/s41467-020-15491-7 (PMC7125299; doi:10.1038/s41467-020-15491-7)
Supplement: Supplementary file 3 — Reporting Summary [file 41467_2020_15491_MOESM3_ESM.pdf]

nature research

Corresponding author(s): Elton P. Hudson

Last updated by author(s): Mar 11, 2020

Reporting Summary

Nature Research wishes to improve the reproducibility of the work that we publish. This form provides structure for consistency and transparency in reporting. For further information on Nature Research policies, see Authors & Referees and the Editorial Policy Checklist .

Statistics

For all statistical analyses, confirm that the following items are present in the figure legend, table legend, main text, or Methods section.

n/a	Confirmed	
☐	☒	The exact sample size (*n*) for each experimental group/condition, given as a discrete number and unit of measurement
☐	☒	A statement on whether measurements were taken from distinct samples or whether the same sample was measured repeatedly
☐	☒	The statistical test(s) used AND whether they are one- or two-sided<br>*Only common tests should be described solely by name; describe more complex techniques in the Methods section.*
☐	☒	A description of all covariates tested
☐	☒	A description of any assumptions or corrections, such as tests of normality and adjustment for multiple comparisons
☐	☒	A full description of the statistical parameters including central tendency (e.g. means) or other basic estimates (e.g. regression coefficient) AND variation (e.g. standard deviation) or associated estimates of uncertainty (e.g. confidence intervals)
☐	☒	For null hypothesis testing, the test statistic (e.g. *F*, *t*, *r*) with confidence intervals, effect sizes, degrees of freedom and *P* value noted<br>*Give P values as exact values whenever suitable.*
☒	☐	For Bayesian analysis, information on the choice of priors and Markov chain Monte Carlo settings
☒	☐	For hierarchical and complex designs, identification of the appropriate level for tests and full reporting of outcomes
☒	☐	Estimates of effect sizes (e.g. Cohen's *d*, Pearson's *r*), indicating how they were calculated

Our web collection on statistics for biologists contains articles on many of the points above.

Software and code

Policy information about availability of computer code

Data collection	For NGS data collection, Illumina BaseSpace (Illumina NextSeq 500) was used. For droplet microfluidics data collection, custom software was used.
Data analysis	-sgRNA reads were mapped to the genome using Bowtie v. 1.2.2<br>-Analysis of depletion/enrichment data from cultivations was done using R v3.6.2 and R topGO v 2.36.0: https://github.com/m-jahn/R-notebooks/tree/master/sgRNA_library<br>-RNA-Seq data was partially analyzed with DESeq2 v 1.22.2 : https://github.com/Asplund-Samuelsson/ribopipe<br>-Creation of sgRNA library was done in Python v 2.7.11. Filtering of NGS data from microfluidics was done using Python v 3.6.4

For manuscripts utilizing custom algorithms or software that are central to the research but not yet described in published literature, software must be made available to editors/reviewers. We strongly encourage code deposition in a community repository (e.g. GitHub). See the Nature Research guidelines for submitting code & software for further information.

Data

Policy information about availability of data

All manuscripts must include a data availability statement. This statement should provide the following information, where applicable:

- Accession codes, unique identifiers, or web links for publicly available datasets
- A list of figures that have associated raw data
- A description of any restrictions on data availability

- NGS data generated in this study, including sgRNA read counts from competition experiments, RNA-Seq data of mutant strains, and sequencing data from sorted droplet microfluidics samples, are provided at the European Nucleotide Archive under accession PRJEB35238 [https://www.ebi.ac.uk/ena/browser/view/PRJEB35238]
- Synechocystis genome reference: NCBI NC_000911.1, [https://www.ncbi.nlm.nih.gov/nuccore/NC_000911.1]

- Figures with associated raw data: Figure 2-6
- No restrictions on availability

Field-specific reporting

Please select the one below that is the best fit for your research. If you are not sure, read the appropriate sections before making your selection.

☒ Life sciences ☐ Behavioural & social sciences ☐ Ecological, evolutionary & environmental sciences

For a reference copy of the document with all sections, see nature.com/documents/nr-reporting-summary-flat.pdf

Life sciences study design

All studies must disclose on these points even when the disclosure is negative.

Sample size	This study was done on bacterial populations, either libraries or clonal populations. Sample sizes were 4 for competition experiments, 3 for RNA seq experiments, and 2 for lactate productivity. Samples size for each experiment was determined based on expected effect size and experimental capacity.
Data exclusions	We did not exclude data
Replication	Growth competition done in 4 replicates. RNA Seq was in 3 replicates. Lactate productivity was in 2 replicates. These data were also reproducible in other cultivation formats. Lactate tolerance was in 2 replicates, and the effects were reproducible in other projects. One cultivation with the mutant strain ilvA was not replicated (Figure 5 C). This is indicated in the Figure legend.
Randomization	Randomization was not performed as all data analysis was done with automated pipelines
Blinding	Blinding was not performed as all data analysis was done with automated pipeline.

Reporting for specific materials, systems and methods

We require information from authors about some types of materials, experimental systems and methods used in many studies. Here, indicate whether each material, system or method listed is relevant to your study. If you are not sure if a list item applies to your research, read the appropriate section before selecting a response.

Materials & experimental systems

n/a	Involved in the study
☒	☐ Antibodies
☒	☐ Eukaryotic cell lines
☒	☐ Palaeontology
☒	☐ Animals and other organisms
☒	☐ Human research participants
☒	☐ Clinical data

Methods

n/a	Involved in the study
☒	☐ ChIP-seq
☒	☐ Flow cytometry
☒	☐ MRI-based neuroimaging
