# Supplementary material for: Pooled CRISPRi screening of the cyanobacterium Synechocystis sp PCC 6803 for enhanced industrial phenotypes
Source: Nat Commun. 2020 Apr 3;11:1666. doi: 10.1038/s41467-020-15491-7 (PMC7125299; doi:10.1038/s41467-020-15491-7)
Supplement: Supplementary file 4 — Description of Additional Supplementary Files [file 41467_2020_15491_MOESM4_ESM.docx]

**Description of Additional Supplementary Files**

**Supplementary Data 1**Text file with gene name, gene locus, sgRNA protospacer sequence, and other regions used for cloning for each sgRNA. (.txt)

**Supplementary Data 2**
Fasta file containing the ncRNA sequences and IDs as represented in Supplementary Data 1. (.fasta)

**Supplementary Data 3**
Summary of all growth conditions, including dilution rates and generation times. (.csv)

**Supplementary Data 4**List of all clones that pass the enrichment threshold in the droplet-based lactate assays and their frequency. (.xlsx)

**Supplementary Data 5**Comparison of *Synechocystis* gene fitness scores with gene essentiality as determined by flux balance analysis of a genome-scale model. (.xlsx)

**Supplementary Data 6**
Comparison of *Synechocystis* gene fitness scores to essentiality of *Synechoccocus* orthologs determined by Tn-Seq. (.xlsx)

**Supplementary Data 7**
Primers used in this study. (.xlsx)
