# Supplementary material for: Pooled CRISPRi screening of the cyanobacterium Synechocystis sp PCC 6803 for enhanced industrial phenotypes
Source: Nat Commun. 2020 Apr 3;11:1666. doi: 10.1038/s41467-020-15491-7 (PMC7125299; doi:10.1038/s41467-020-15491-7)
Supplement: Supplementary file 2 — Peer Review [file 41467_2020_15491_MOESM2_ESM.pdf]

Reviewers' comments:

Reviewer #1 (Remarks to the Author):

In this manuscript Yao et al. perform a pooled CRISPRi screens of Synechosystis. Pooled CRISPRi screens are a powerful approach to unravel gene function that presents an interesting alternative to transposon based screens. Similar CRISPRi screens have already been performed in E. coli, B. subtilis and Vibrio natriegens, but this is the first application of this strategy to cyanobacteria to our knowledge. By constructing a genome-wide library (2 sgRNA/gene), the authors investigated the fitness contribution of all Synechocystis annotated genes to cellular fitness and growth rate in a set of conditions including different illuminations and conditions to assess L-lactate tolerance. This set of experiments recapitulated previously known results, validating the method and experimental design used, but also provides a number of novel gene x phenotype relationships.

Finally, the sgRNA library was cloned in a L-lactate producing strain. Microfluidics combined to fluorescence-based L-lactate assay enriched sgRNAs enhancing production of this chemical. This assay is particularly interesting as it involves a microfluidic setup to capture a phenotype that could not be accessed in a pool. As discussed by the authors this microfluidic setup did not perform as well as might have been expected, but the authors were still able to obtain meaningful information from the assay and propose directions to improve it.

Altogether an impressive amount of work is presented in a manuscript that is well written. This is a very nice application of the CRISPRi screen technique, which led to interesting and novel insights into Synechocystis biology. The problems have been thoroughly investigated, experimental design appeared well-thought, and technical realization well executed. I would like to congratulate the authors for this admirable work. I find it impressive to have a library size of ~10k sgRNAs in a cyanobacteria, in particular considering the difference in growth rate with heterotrophs such as E. coli. That being said, the limited library size results in trade-offs in genome coverage vs redundancy. The authors favoured the former at the expense of the latter. For a first CRISPRi screen, a more redundant library design could have been adopted to better control for differences between sgRNA repression efficiencies. This would have helped understand sgRNA design rules for this bacteria.

I would also like to congratulate the authors on making the effort to provide a nice web interface to explore the data.

Comments:

No mention is made of polar effects. How were they taken into account in the library design and data analysis? This is an important point as dCas9 will block the expression of all downstream genes in an operon.

Some definitions, methods or pieces of information are missing or imprecise. The most prominent example is probably how the author go from observations on the sgRNA level to observations on the gene level. This is not explained in the current manuscript and is clearly lacking. Details about the L-lactate producing library are similarly lacking. Finally, not all scripts used in this study have been made publicly available, which would have helped precisely understand what processing/analysis were performed.

Minor comments:

Please specify which dCas9 variant is used in the study.

“Interestingly, nearly all genes with a low fitness score are also among proteins found by Jahn et al. to be regulated with growth rate (Supplemental Fig. 1).”
Until this point, you were discussing sgRNA fitness. Please explain briefly how you obtained gene fitness from these data.

“the class-II FBP/SBP aldolase gene (cbbA, sll0018), which is needed to synthesize sedoheptulose bisphosphate (SBP) for both routes, was essential.”
You do not provide a clear definition of gene essentiality in your setup. Is it when both sgRNA targeting the gene are depleted beyond a given threshold (what threshold?)? Or when only one of the two is depleted? Is it when sgRNAs are depleted in all tested conditions or only one? …

“There were 25 genes with differential fitness between L100 and LD, and only 4 between L300 and LD”
Could you briefly develop on these, as you did for genes with a differential fitness between L100 and L300? What pathway(s) do these genes correspond to? How do the 25- and 4-genes sets compare to each other, are the four genes included in the 25?

“From the RNA-Seq data, slr1916 and ssl2982 mutants had 143 and 248 differential transcripts compared to a control strain”
You mean “differentially expressed”, reformulate.

“An upregulation of carotenoid biosynthesis suggests increased PSII repair.”
As no PSII protein is upregulated in these transcriptomic analyses, I find the claim a bit bold. It is also contradicted by the RpaB result described a few lines latter: “Interestingly, the high-light responsive transcription factor RpaB (slr0947, regulator of phycobilisome association B) was also up-regulated in multiple mutants. The extensive regulon of RpaB includes likely repressor activity of linear electron transport at high light (e.g. subunits of PSII and Cyt-b6f)”

“We found 78 sgRNAs that were enriched during the L-lactate cultivation, but not in a NaCl control cultivation (Fig. 5a). Eight genes were enriched with both sgRNAs (Fig. 5b). Curiously, 19 of the enriched sgRNAs (24%) targeted genes in amino acid metabolism and protein biosynthesis, including multiple aminoacid tRNA synthetases (argS, aspS, asnS, glyS, gltX, metS) (Supplemental Fig. 6).”
Supp. Fig. 6 represents 59 sgRNAs. Fig. 5b represents 8 genes with both sgRNAs enriched, so 16 sgRNAs. Either 3 sgRNAs (78-(59+16)) are lacking on Supp. Fig. 6, or only 75 sgRNAs are enriched during L-lactate cultivation.

Contrary to the WT library, you do not provide any information on the L-lactate library. As for the WT, please indicate (in the result or method section):
- how many clones you collected;
- how much sequencing you performed;
- if you performed the analysis in the same way, if not please provide more details about the differences;
- how many sgRNA you detected in this second library;
- the library coverage;
- etc…

"This criterium returned 528 clones, 359 harbored sgRNAs targeting ORFs and 169 harbored sgRNAs targeted to ncRNAs (Supplemental Data 8).”
Please detail how many sgRNAs appear in the top 10% in all 4, in 3 and in 2 sorted samples,

especially as Table 1 only presents a subset of these sgRNAs.

"In an attempt to uncover common regulations among these mutants, we reconstructed slr1916, sll1969, slr1340, and ssl2982 sgRNA mutants and collected transcriptomics after CRISPRi induction." Do you mean that you constructed deletion mutants or that you cloned the guides targeting these genes? I wouldn't use the word "mutant" for the latter.

"slr1340, an uncharacterized gene with encoding a predicted acetyl-transferase"
Please rephrase.

"The library was grown in shake-flasks, gene repression was induced, and samples were taken after 36 and 66 h to assay for L-lactate productivity "
A few more details in the main text would be welcome here. You should at least specify that a fluorescence assay is used and that pico-injection is involved.

"The dynamic nature of CRISPRi repression could cloud beneficial effects"
Please rephrase.

I find a bit surprising to develop 6 full lines on the LI-POR enzyme in the discussion. This represents 74 words on the 880 of the discussion, for a result not directly related to the main point of this paper, and representing only 1 line of the result section.
I would prefer other results to be more extensively discussed.

"The pooled oligos were cloned into a genome targeting vector (locus slr039774, kanamycin resistance) containing the PL22 promoter by Golden Gate assembly."
Please specify the vector used. If not readily available, please consider depositing it into vector repositories or make a statement about plasmid distribution.

Please explicitly describe sgRNA naming convention.

Reference genome ID is wrong. Likely correct ID is NC_000911.1
https://www.ncbi.nlm.nih.gov/nuccore/NC_000911.1

sgRNA location in Supp. Data 1 do not agree with the provided sgRNA sequence.
For first sgRNA:
>sll1336|66|1|[[3287121, 0]]
CAGTGATAGAGATACTGGGAGCTATTTGTGGATATTGCCCTCCATCCAGTTTTAGAGCTAGAAATAGCAAGTTAAAAT

Highlighted sequence position on NC_000911.1 is 3287076-3287099, off by -22 from indicated 3287121 position. No canonical SpCas9 PAM can be found next to position 3287121.

Please indicate the cultivation temperature for all experiments

"Anhydrotetrocycline inducer was added to 500 ng/uL to the culture"
"aTc inducer (added to 500 ng/mL)"
Please correct the wrong one

"Mutants were pre-cultivated in BG-11 with antibiotics (25 µg/mL spectinomycin, 25 µg/mL kanamycin)"
Were the screen cultures performed with antibiotics? If so, please include antibiotics used and

concentration.

“Determination of fold change and significance was based on aggregating 4 independent biological replicates for all cultivations.”
Please explicitly state what you mean by “aggregating”.

“To cluster genes based on depletion/enrichment pattern, a dissimilarity matrix was computed using R’s dist function with distance measure euclidean.”
Fig. 2 represents sgRNA clustering, not gene clustering. The Results section also mentions sgRNA clustering and not gene clustering.
On what data was the clustering performed?

“Silhouette analysis was performed to find the optimal number of clusters (silhouetteAnalysis from package silhouette) and showed equally good separation for 3 to 9 clusters. A number of 5 clusters was chosen representing sgRNAs with decreasing level of depletion (1-4) as well as unchanged sgRNAs (5).”
The clusters reported in Fig. 2 do not match the reported clustering. Indeed, the highest split in the clustering tree is (clusters 1 to 4) vs cluster 5.
Second highest split would separate cluster 5 in two.
Third split separates cluster 1+2 vs cluster 3+4.
Fourth is cluster 3 vs cluster 4.
Fifth is cluster 1 vs cluster 2.
With the reported clustering, if clusters 1, 2, 3 and 4 are separated, then cluster 5 should be split in two clusters, one with sgRNA increasing Log2FC in LD300 (+ and – conditions), corresponding to ~1/4 of all sgRNA, and one containing sgRNA with small changes in Log2FC.
Either the clustering reported in Fig. 2 is not the correct one, or the 5 clusters are not based on the clustering. Please correct Fig. 2 with the clustering actually used, or split cluster 5 in two as the clustering indicates.

“To find genes involved in L-lactate tolerance, sgRNAs enriched specifically for presence of sodium L-lactate but not sodium chloride were selected (threshold: log2 FC ≥ 2, -log10 p-value ≥ 20). Of the 78 sgRNAs falling into this category, none was enriched under sodium chloride”
Either the first sentence should be “To find genes involved in L-lactate tolerance, sgRNAs enriched specifically for presence of sodium L-lactate but not sodium chloride were selected (threshold: log2 FC ≥ 2, -log10 p-value ≥ 20).”, or the second phrase is redundant.

“Using NGS obtained on the sorted droplets, that approximately 8,000 unique sgRNAs were detected from approximately 5 million mapped reads.”
Unclear sentence, please rephrase.

“Next, sgRNAs with a 10-fold difference in enrichment factor between replicates were removed.”
“≥ 10-fold”?

For all dot plots, please consider adding transparency to better visualize superimposed individual dots as in Fig. 2c. Concerned figures are Fig. 3a, 4b, 5a, as well as Supp. Fig. 3a, 4a, and most importantly Supp. Fig 10

Reviewer #2 (Remarks to the Author):

Review on manuscript NCOMMS-19-35755
"Pooled CRISPRi screening of the cyanobacterium Synechocystis sp. PCC 6803 for enhanced growth,

tolerance, and chemical production"

Cyanobacteria play important roles in many aquatic and terrestrial environments, as primary but also as toxin producers. In addition to their global ecological role, cyanobacteria recently came also in the focus as green cell factories to establish CO2-neutral biosynthesis of different products. Despite many promising examples for proof of concept, the production titers are mostly low, which is certainly related to our lack of knowledge on cyanobacterial regulation mechanisms for growth and for metabolism. The CRISPRi technique is a powerful tool to target many or even all genes in a bacterium and subsequently analyze the effects on different cellular levels. The authors showed here the genome-wide CRISPRi application towards the model cyanobacterium Synechocystis sp. PCC6803, which is often used in the basic and applied research of many different aspects among cyanobacteria. The authors screened the CRISPRi libraray for genes potentially limiting growth of Synechocystis or have impact on L-lactate production with this strain. They present lists of genes that were depleted of enriched in the sgRNA mutant library under different growth conditions. In some cases, they started also attempts to verify the role of identified genes by single CRISPR-induced gene silencing approaches. These approaches showed that the initially identified genes seem to play a role in the investigated traits, but more work is necessary.

Major points
1. The real efficiency of the CRISPRi attempt is not really clear. The authors mention that the obtained 300,000 Synechocystis transformants, which were pooled and used for the later experiments. Please show data, how many of the initially synthesized sgRNAs were really included in the libraries, which were used for the three different applications. Did you observe cases that more than one sgRNA construct was found in one Synechocystis clone?
2. The authors are very confident. I think the CRISPRi screening only identifies genes that are potentially/possibly related to growth limitation or lactate productivity. Enrichment or depletion could also result from indirect effects. Therefore, I would suggest to weaken the statements and to improve discussion on advantages but also problems with this approach.
Specific points:
1. Second line abstract: The data presented in the manuscript clearly show that not all genes were really targeted, please mention that you aimed to target all genes!
2. Second last sentence of introduction: What means “biochemical production”?
3. End of first para Results: As mentioned above, it is essential to show the data how representative your library was.
4. Second para: please mention the light or dark periods, e.g. 12/12 h
5. Second para: 7119 sgRNA clones were investigated. This corresponds to about 60% of the initial library, what does this mean, did you found these 7119 sgRNAs clones in each treatment, i.e. are in each treatment always the same sgRNAs missing?
6. Second para: summing up cluster 1-4 results in 1992 and not as mentioned above 1998,
7. Second para: summing up clusters 1-5 results in 7113 sgRNAs and not 7119 mentioned above
8. End of second para: how good is the overlap to essential genes identified in the transposon-based screen in S. elongatus?
9. Page 5, last para: he authors discuss two possibilities why normal light and high light data vary. I suggest a third explanation, which is related to light/oxidative stress induction in the high light cultures. These cells are probably already more stressed than cells at 100 micro Einstein leading to the severe down-regulation of key enzymes? This assumption is well supported by genes found in cluster 3, many of them known to be crucial for high light/redox stress acclimation.
10. Page 5, last sentence: The lower effect of L/D growth on essential genes could be also related to the fact that S. elongatus has a much higher percentage of strictly circadian-regulated genes than 6803?
11. Page 6: The finding of omega subunit is remarkable, since the mutant is impaired for example in carbon acclimation. May be that this point could be discussed.
12. Page 7, first para: The upregulation of carotenoid synthesis genes is attributed to PSII repair. Why? Direct evidence for enhanced PSII repair would be expression stimulation of genes for different

D1 protein genes or FtsH genes. Probably more carotenoids simply show a general acclimation to light stress.
13. Page 7, first para: ndhD1 and ndhD2 are not crucial for CO2 uptake but more specifically related to cyclic electron flow!
14. Page 7 first para: The differential regulation of rpaB in the selected sgRNA mutants best explains why many genes related to photosynthesis are changed in this mutant set. I would start with this finding and then discuss the other findings in relation to the effect of this or possibly some other regulatory proteins.
15. Page 7, second para: (up to 15 mM)
16. Page 7, second para: The authors mention that many clones had growth defects in the absence of L-lactate. Please show these data!
17. Page 8, first sentence: The assumption that certain amino acids may have specific protective effects can be easily tested, just by addition of single amino acids to the L-lactate medium, since for most of them uptake systems exist in 6803!
18. Supp.Fig. 7: I did not see growth improvement for all repression clones in Suppl. Fig. 7, please show delta growth of repression strains minus growth of control mutant. The growth oscillations are very surprising. After my opinion the oscillation are not very likely due to circadian rhythm. For Synechocystis never long free-running rhythms have been shown! May be that the rhythm was induced by lactate additions? it is also not obvious for all growth curves!
19. Fig. 5: if I understood the symbols in the figure right, only two repression clones show significantly improved growth! or is the growth of all light grey columns significantly improved by $p = 0.05$? This is unlikely at least for the asd gene. Please refer only to genes that were verified by statistical significant differences.
20. Page 8, end of first para: After my opinion, the improved pigmentation simply shows less toxic effects of L-lactate.
21. Page 8, last para: again numbers are missing, how representative the library really was!
22. Page 9, first para: The authors refer to genes that were found in two out of four attempts. Is that statistical significant? how many genes were reproducibly found in three or even four sorted samples?
23. Page 9, middle para: The ATP/NADPH ratio is discussed as main possibility to improve production of certain biochemical. Then, two sgRNA clones are mentioned that possibly have enhanced ATP levels. Did you try to measure the ATP amounts to support this speculation?
24. Table 1: I would expect at least some overlap with genes that were identified in the screening for L-lactate resistant clones, why is there nothing?
25. Figure 6: from the data in Fig. 6 it is not clear which differences at the last sampling point are statistically significant, please include this information!
26. Discussion, third line: how cyanobacteria POTENTIALLY solve…
27. Page 11, end: 87% overlap is mentioned to FBA, this overlap is surprisingly high, since the model approach can only include annotated genes, whereas here often genes were found coding for proteins of unknown or hypothetical proteins, which are certainly not in the FBA. In the clusters mentioned above about 1900 genes were involved, where the number 329 comes from?
28. Fig. 6a: colors difficult to distinguish

Reviewer #3 (Remarks to the Author):

Review fo Yao2019 NCOMMS-19-35755

- First time genome-scale essentiality data. Important and useful technological achievement. Identified several "mutants" with enhanced biotechnological properties, several of which were also verified independently by reconstruction.

- Of major concern is the lack of accessibility in the figures, in particular. Not only are there too many unneccessary abbreviations, it is often impossible to understand what the point of any one figure

panel from the figure itself, even when reading the legend.

- The second concern is the apparent lack of data from diurnal conditions even though some early experiments did include such conditons - why not report on the data from them as surely it must have been collected?

- The verification of the mutants with improved growth rate is unclear or missing (difficult work out which)?

- Overally, the writing is often sloppy without consideration of intellectual interest for the reader. The data is actually quite interesting and there is more to discuss than presented. In addition, there is considerable discussion in the results section (e.g. page 6), why not just combine the two?

Improvements
- Line numbers are essential for an effective review process

- Abstract is unclear, 3rd to last sentence. 2nd to last sentence is even more unclear, perhaps due to a word limit? What caused what? % what?

- Introduction, the need in general by the research field is not specifically essentiality, but rather better understanding on how the system works as a whole. Essentiality is part of that but not the only knowledge we need. How many genes in 6803 have a verified function relative to E. coli, for example? Some context would be useful.

- 'both photoautotrophic and diurnal growth' is unclear? When is the latter not also the former?

- Does '>10,000' = 'all annotated genes' = 'all open reading frames'? The start of the Results section (no line numbers provided) suggest they are not the same? How many genes are annotated in 6803 and where was that done, i.e. on what basis?

- Similarly, 2 x each gene => 10,498 sgRNA sequences => Less than 10,000 genes???

- 'several knockdowns.'feels sloppy'

- Overall, the introduction feels like a collection of separate items rather than a smooth-flowing story. Why is this work of interest other than just technological achievement?

- Last sentence in first paragraph of Results section is poorly written.

- Was there any contamination of the cultures? Was this checked for in any way?

- What is 'depletion pattern'?

- 'We examined genes in central carbon metabolism for effects of CRISPRi repression'. Why? How?

- 'A second explanation is that faster growing cells are more prone to perturbation of enzyme levels, due to higher enzyme saturation' is unclear

- First paragraph in 'mutants with a growth advantage'. What was the outcome from diurnal conditions? For example, was the outcome similar for calvin cycle and photorespiration related genes? That would be more interesting in relation to fundamental biology than constant light.

- Were the mutants with increased specific growth rate confirmed separately as was done with the mutants identified to have improved growth in the presence of lactate? I was not able to see this and

the conclusion on this part of the work was muddy.

- Discussion. How conditional is the outcome? What if the expression of the sgRNA varied, for example? Surely this must have been optimized at some point of the study?

- How can we interpret this data in the context of evolution? The conditions were highly specific, yet evolution most likely has favoured robustness over specific growth rate speed? It would be interesting to see some discussion on this even if speculative.

- Figures. There is no need to abbreviate everything. FC, dF, F, it's too much. Spell it out instead. Similar for NACL and LAC, why not use much more recognisable words? NaCl, or Lactate?

- The majority of the figure panels are impossible to follow though I imagine a specialist working with similar methods will understand. Should the results be accessible to all readers or only some?

4C, what do the colours mean? There is no explanation in the panel. There is space to describe it in the figure rather than just in the legend.

4D what is the relationship between the genes written over the grey background at the top of each sub-section and those within the rectangles?

5E show the difference to wild-type instead, a large number of very similar spectra with very similar colours are practically impossible to decipher.

Fig. 6 would benefit from statistical analyses in the figure rather than just error bars.

Reviewer response document

Yao et al., **NCOMMS-19-35755**

Reviewer #1 (Remarks to the Author):

In this manuscript Yao et al. perform a pooled CRISPRi screens of Synechosystis. Pooled CRISPRi screens are a powerful approach to unravel gene function that presents an interesting alternative to transposon based screens. Similar CRISPRi screens have already been performed in E. coli, B. subtilis and Vibrio natriegens, but this is the first application of this strategy to cyanobacteria to our knowledge. By constructing a genome-wide library (2 sgRNA/gene), the authors investigated the fitness contribution of all Synechocystis annotated genes to cellular fitness and growth rate in a set of conditions including different illuminations and conditions to assess L-lactate tolerance. This set of experiments recapitulated previously known results, validating the method and experimental design used, but also provides a number of novel gene x phenotype relationships.

Finally, the sgRNA library was cloned in a L-lactate producing strain. Microfluidics combined to fluorescence-based L-lactate assay enriched sgRNAs enhancing production of this chemical. This assay is particularly interesting as it involves a microfluidic setup to capture a phenotype that could not be accessed in a pool. As discussed by the authors this microfluidic setup did not perform as well as might have been expected, but the authors were still able to obtain meaningful information from the assay and propose directions to improve it.

Altogether an impressive amount of work is presented in a manuscript that is well written. This is a very nice application of the CRISPRi screen technique, which led to interesting and novel insights into Synechocystis biology. The problems have been thoroughly investigated, experimental design appeared well-thought, and technical realization well executed. I would like to congratulate the authors for this admirable work. I find it impressive to have a library size of ~10k sgRNAs in a cyanobacteria, in particular considering the difference in growth rate with heterotrophs such as E. coli. That being said, the limited library size results in trade-offs in genome coverage vs redundancy. The authors favoured the former at the expense of the latter. For a first CRISPRi screen, a more redundant library design could have been adopted to better control for differences between sgRNA repression efficiencies. This would have helped understand sgRNA design rules for this bacteria.

I would also like to congratulate the authors on making the effort to provide a nice web interface to explore the data.

Comments:

No mention is made of polar effects. How were they taken into account in the library design and data analysis? This is an important point as dCas9 will block the expression of all downstream genes in an operon.

The reviewer raises a very important point for CRISPRi. We did not consider polar effects when designing the sgRNA library, as we designed sgRNAs to bind every gene, regardless of operon position. Regarding data analysis, we do not think polar effects should complicate functional clustering, as often functionally related genes are in an operon. However, polar effects can complicate interpretation of individual gene fitness.

We have taken the reviewer's suggestion and expanded on polar effects by adding new text to the Discussion section. Here we also stress that fortunately there is detailed mapping of operons in Synechocystis (these are also annotated in the reference genome). We also give an example of how polar effects can complicate interpretation of library data (Line 356):

> "Care should be taken when using results from CRISPRi libraries to determine gene essentiality. We used 2 sgRNAs targeting each ORF; recent studies have shown that more are needed to ensure statistical significance when assessing gene essentiality[11]. Furthermore, a confident determination of gene essentiality from CRISPRi data would require consideration of a gene's place in an operon. Data from *E. coli* CRISPRi libraries showed a strong polar effect, where targeting the first gene in an operon also represses downstream genes, while the reverse polar effect, i.e. repression of downstream genes affecting expression of upstream genes, was not prominent (Cui et al., 2018). For *Synechocystis*, transcription start sites have been mapped in detail for many conditions and are annotated in the reference genome (NC_000911.1; (Kopf et al., 2014)). Therefore, fitness data for individual genes can be considered with their operon context. For example, the *pyk2* gene *(sll1275)* encoding pyruvate kinase 2 is at the 5' end of an operon that also contains *sll1276* (probable iron transporter) and *recF* downstream. In our data, both sgRNA clones targeting *pyk2* showed severe growth defects, suggesting it is important for cell growth. However, one sgRNA clone targeting *sll1276* also shows a strong growth defect (neither clone targeting *recF* shows a growth defect). Considering previous attempts to knock out *sll1276* were unsuccessful, it is likely that *sll1276* is essential for cell growth. Based on the sgRNA data alone, we cannot unambiguously conclude that *pyk2* is essential for growth, as the downstream *sll1276* is also likely repressed in those clones."

Fortunately, the operon structure of Synechocystis has been described through experimental determination of all transcription start sites in various conditions (Kopf et al., 2014 doi: 10.1093/dnares/dsu018). This resource will allow users of our data to check the operon structure of their gene of interest. The reviewer is correct, that repression of the first gene in an operon can give a false positive if a downstream gene is critical.

We have edited the Discussion to add operon structure as a caveat of using CRISPRi, particularly for using it to assign gene essentiality.

Some definitions, methods or pieces of information are missing or imprecise. The most prominent example is probably how the author go from observations on the sgRNA level to observations on the gene level. This is not explained in the current manuscript and is clearly lacking. Details about the L-lactate producing library are similarly lacking. Finally, not all scripts used in this study have been made publicly available, which would have helped precisely understand what processing/analysis were performed.

We fully agree that the separation between sgRNA level and gene level was not entirely clear. Our study focuses mostly on the sgRNA level but occasionally 'summarizes' the results of the two sgRNAs of a particular gene. As detailed below, we have now addressed all specific issues raised by the reviewer and hope that usage of sgRNA or gene level is now clear throughout the manuscript. We also included explicit statements in the Results of how fitness of 2 sgRNAs was summarized to obtain gene level fitness.

Regarding script availability, all statistical analyses are publicly available and reproducible through the provided data and github repositories. We now include links to these in the Methods section (ex L481).

Minor comments:

Please specify which dCas9 variant is used in the study.
The dCas9 information was added in the Methods section: "A catalytically dead Cas9 (dCas9, mutations D10A and H840A) from Streptococcus pyogenes was used for gene repression in this study"

"Interestingly, nearly all genes with a low fitness score are also among proteins found by Jahn et al. to be regulated with growth rate (Supplemental Fig. 1)."
Until this point, you were discussing sgRNA fitness. Please explain briefly how you obtained gene fitness from these data.

The reviewer correctly points out that information is missing on how gene-level fitness was obtained from sgRNA fitness. The original phrase is incorrect in the sense that gene fitness was

not used at this point. What is shown instead are sgRNA fitness scores. Both sgRNAs were mapped to their corresponding target protein, so that every unique protein in Suppl. Fig. 1 (here represented as ANOVA p-value) has two corresponding unique sgRNA fitness scores. We corrected the paragraph, L116

"the class-II FBP/SBP aldolase gene (cbbA, sll0018), which is needed to synthesize sedoheptulose bisphosphate (SBP) for both routes, was essential."
You do not provide a clear definition of gene essentiality in your setup. Is it when both sgRNA targeting the gene are depleted beyond a given threshold (what threshold?)? Or when only one of the two is depleted? Is it when sgRNAs are depleted in all tested conditions or only one? …

We have generally assumed that if 1 of the 2 sgRNAs have a strong fitness effect then the effect is real. However, as we discuss at the end of the paper, more sgRNAs per gene are likely needed to make comments on essentiality, due to variability in CRISPRi repression, as well as potential operon effects.

We have now refrained from using essentiality when referring to sgRNA data, instead "strong fitness effect." We do use "essentiality" when referring to data from transposon insertion studies (such as Rubin et al., 2015), where essentiality was deduced from absence of insertion mutants in a gene, where otherwise genes had multiple transposon insertions.

"There were 25 genes with differential fitness between L100 and LD, and only 4 between L300 and LD"
Could you briefly develop on these, as you did for genes with a differential fitness between L100 and L300? What pathway(s) do these genes correspond to? How do the 25- and 4-genes sets compare to each other, are the four genes included in the 25?

Based on the criteria that we used to select genes with differential fitness, there was no overlap of the selected genes from L300 vs. LD compared to L100 vs. LD (see Venn diagram below). However, genes with differential fitness at the L100-L300 comparison overlapped partially with genes from L300-LD (2 out of 5) and L100-LD (15 out of 22). We can conclude from this comparison that gene fitness is very similar between conditions L300 and LD, while it is more dissimilar for L100 compared to L300 or LD. We expanded our discussion of this result in the manuscript, L157-159.

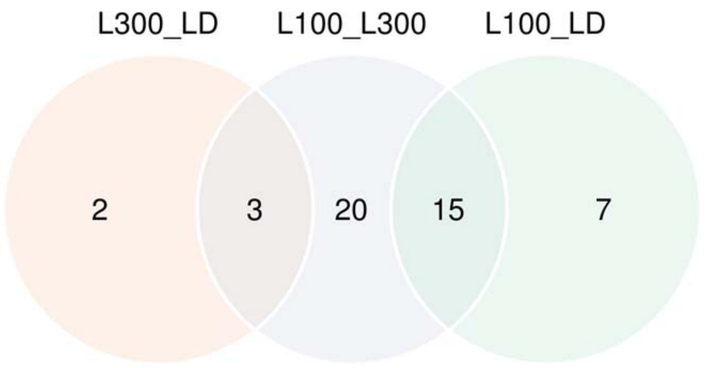

“From the RNA-Seq data, slr1916 and ssl2982 mutants had 143 and 248 differential transcripts compared to a control strain”
You mean “differentially expressed”, reformulate.
Corrected according to reviewers suggestion.

“An upregulation of carotenoid biosynthesis suggests increased PSII repair.”
As no PSII protein is upregulated in these transcriptomic analyses, I find the claim a bit bold. It is also contradicted by the RpaB result described a few lines later: “Interestingly, the high-light responsive transcription factor RpaB (slr0947, regulator of phycobilisome association B) was also up-regulated in multiple mutants. The extensive regulon of RpaB includes likely repressor activity of linear electron transport at high light (e.g. subunits of PSII and Cyt-b6f)”
We have toned down our claims regarding PSII repair

“We found 78 sgRNAs that were enriched during the L-lactate cultivation, but not in a NaCl control cultivation (Fig. 5a). Eight genes were enriched with both sgRNAs (Fig. 5b). Curiously, 19 of the enriched sgRNAs (24%) targeted genes in amino acid metabolism and protein biosynthesis, including multiple aminoacid tRNA synthetases (argS, aspS, asnS, glyS, gltX, metS) (Supplemental Fig. 6).”
Supp. Fig. 6 represents 59 sgRNAs. Fig. 5b represents 8 genes with both sgRNAs enriched, so 16 sgRNAs. Either 3 sgRNAs (78-(59+16)) are lacking on Supp. Fig. 6, or only 75 sgRNAs are enriched during L-lactate cultivation.
The reviewer is correct. The number of 78 sgRNAs that is found in the text and Figure 5 a is incorrect. The mistake was caused by falsely counting also NA (not available) values in Figure 5 a's upper right quadrant. Correct is that 75 sgRNAs were enriched, the number that was also used in subsequent plots. The manuscript L228 and Figure 5 a were corrected.

Contrary to the WT library, you do not provide any information on the L-lactate library. As for the WT, please indicate (in the result or method section):
- how many clones you collected;
- how much sequencing you performed;

- if you performed the analysis in the same way, if not please provide more details about the differences;
- how many sgRNA you detected in this second library;
- the library coverage;
- etc…

We took the reviewer's advice and included the information in the results section (L263). In the Methods, we now clarify that the methodology to create the lactate library was essentially the same as to the WT library. In the results section, we also point out that 10,495 sgRNAs could be detected in the lactate library.

"This criterium returned 528 clones, 359 harbored sgRNAs targeting ORFs and 169 harbored sgRNAs targeted to ncRNAs (Supplemental Data 8)."
Please detail how many sgRNAs appear in the top 10% in all 4, in 3 and in 2 sorted samples, especially as Table 1 only presents a subset of these sgRNAs.

We have removed the statement "top 10%". We have now made a few changes in text to clarify our analysis. We state that for each sorted sample, we compute the ratio of sorted/unsorted clones (in abundance). At this stage, we counted approximately 1600, 3000, 5300 and 4700 unique clones in each sorted sample. We applied a data filter to return only clones with a sorted/unsorted ratio greater 3. After this filter, we obtained approximately 600, 900, 850, and 600 clones from each sample. L280-286

"In an attempt to uncover common regulations among these mutants, we reconstructed slr1916, sll1969, slr1340, and ssl2982 sgRNA mutants and collected transcriptomics after CRISPRi induction."
Do you mean that you constructed deletion mutants or that you cloned the guides targeting these genes? I wouldn't use the word "mutant" for the latter.
We cloned the corresponding sgRNAs in a dCas9 background strain. We followed the reviewer's suggestion to make clear that these are not deletion mutants.

"slr1340, an uncharacterized gene with encoding a predicted acetyl-transferase"
Please rephrase.
Corrected.

"The library was grown in shake-flasks, gene repression was induced, and samples were taken after 36 and 66 h to assay for L-lactate productivity "
A few more details in the main text would be welcome here. You should at least specify that a fluorescence assay is used and that pico-injection is involved.
We added text in the Results describing the droplet assay, in particular stating that we did picoinjection

“The dynamic nature of CRISPRi repression could cloud beneficial effects”
Please rephrase.
To improve clarity we rephrase this to:
"The variability in CRISPRi repression efficiency and in sensitivity of a gene towards repression could lead to false negative observations (i.e. knockdown of a gene does not improve tolerance because repression level is not optimal)."

I find a bit surprising to develop 6 full lines on the LI-POR enzyme in the discussion. This represents 74 words on the 880 of the discussion, for a result not directly related to the main point of this paper, and representing only 1 line of the result section.
I would prefer other results to be more extensively discussed.
We have removed some of the text around Li-POR.

“The pooled oligos were cloned into a genome targeting vector (locus slr039774, kanamycin resistance) containing the PL22 promoter by Golden Gate assembly.”
Please specify the vector used. If not readily available, please consider depositing it into vector repositories or make a statement about plasmid distribution.
More detailed information about this plasmid was added to Methods (L442). We have also made a statement about accessibility of the strains and plasmids, that they are freely available from us upon request (L460).

Please explicitly describe sgRNA naming convention.
This has been added to Supplemental Data 1, please see comments below.

Reference genome ID is wrong. Likely correct ID is NC_000911.1
https://www.ncbi.nlm.nih.gov/nuccore/NC_000911.1
The reference genome ID was corrected.

sgRNA location in Supp. Data 1 do not agree with the provided sgRNA sequence.
For first sgRNA:
>sll1336|66|1|[[3287121, 0]]
CAGTGATAGAGATACTGGGAGCTATTTGTGGATATTGCCCTCCATCCAGTTTTAGAGCTAG
AAATAGCAAGTTAAAAT

Highlighted sequence position on NC_000911.1 is 3287076-3287099, off by -22 from indicated 3287121 position. No canonical SpCas9 PAM can be found next to position 3287121.

We took the reviewer’s advice and changed Supp. Data 1. Since the location of sgRNAs binding within the ORF is already given as the second number in the index (position is 66 in the example provided above), we removed the numbers in brackets. We also added information on the index naming convention in Supp. Data 1.

Please indicate the cultivation temperature for all experiments
We added cultivation temperatures in the methods section.

“Anhydrotetrocycline inducer was added to 500 ng/uL to the culture”
“aTc inducer (added to 500 ng/mL)”
Please correct the wrong one
Corrected, it should read 500 ng/mL

“Mutants were pre-cultivated in BG-11 with antibiotics (25 µg/mL spectinomycin, 25 µg/mL kanamycin)”
Were the screen cultures performed with antibiotics? If so, please include antibiotics used and concentration.
We used the same concentration of antibiotics for the screen cultivation in the photobioreactor. Information was added in the methods section.

“Determination of fold change and significance was based on aggregating 4 independent biological replicates for all cultivations.”
Please explicitly state what you mean by “aggregating”.
Rephrased for clarity: "For each screened condition, fold changes and p-values were calculated from averaging 4 independent biological replicate cultivations" (L564)

“To cluster genes based on depletion/enrichment pattern, a dissimilarity matrix was computed using R’s dist function with distance measure euclidean.”
Fig. 2 represents sgRNA clustering, not gene clustering. The Results section also mentions sgRNA clustering and not gene clustering.
On what data was the clustering performed?
The reviewer is correct, clustering was performed on sgRNAs, not genes. The mistake was corrected (Line 577).

“Silhouette analysis was performed to find the optimal number of clusters (silhouetteAnalysis from package silhouette) and showed equally good separation for 3 to 9 clusters. A number of 5 clusters was chosen representing sgRNAs with decreasing level of depletion (1-4) as well as unchanged sgRNAs (5).”
The clusters reported in Fig. 2 do not match the reported clustering. Indeed, the highest split in the clustering tree is (clusters 1 to 4) vs cluster 5.
Second highest split would separate cluster 5 in two.
Third split separates cluster 1+2 vs cluster 3+4.
Fourth is cluster 3 vs cluster 4.
Fifth is cluster 1 vs cluster 2.
With the reported clustering, if clusters 1, 2, 3 and 4 are separated, then cluster 5 should be split in two clusters, one with sgRNA increasing Log2FC in LD300 (+ and – conditions), corresponding to ~1/4 of all sgRNA, and one containing sgRNA with small changes in Log2FC. Either the clustering reported in Fig. 2 is not the correct one, or the 5 clusters are not based on the clustering. Please correct Fig. 2 with the clustering actually used, or split cluster 5 in two as the clustering indicates.

The reviewer correctly points out that cluster 5 consists of 2 sub-clusters. This was not a mistake but intentionally done in order to group all unchanged sgRNAs in one cluster for the sake of simplicity (this group is omitted from further analysis in the following parts of the manuscript). The reason to do so was that unsupervised clustering actually produces 6 clusters, not 5, as the reviewer noticed. However, although these two clusters contain mostly unchanged sgRNAs, they are separated by a group of sgRNAs (around 25% of all) that show a spurious, temporary enrichment for 2 specific time points (1 and 2 day measurements). We assume that this temporary enrichment was due to technical variation (e.g. in sample preparation) and has no biological explanation. We added this information to the corresponding results and methods sections (L109-112; L577), and updated Figure 2.

"To find genes involved in L-lactate tolerance, sgRNAs enriched specifically for presence of sodium L-lactate but not sodium chloride were selected (threshold: log2 FC ≥ 2, -log10 p-value ≥ 20). Of the 78 sgRNAs falling into this category, none was enriched under sodium chloride"
Either the first sentence should be "To find genes involved in L-lactate tolerance, sgRNAs enriched specifically for presence of sodium L-lactate but not sodium chloride were selected (threshold: log2 FC ≥ 2, -log10 p-value ≥ 20).", or the second phrase is redundant.
The redundant phrase was removed.

"Using NGS obtained on the sorted droplets, that approximately 8,000 unique sgRNAs were detected from approximately 5 million mapped reads."
Unclear sentence, please rephrase.
Rephrased to "Using NGS obtained on each sorted droplet fractions, we could map 5 million reads per sample to approximately 8,000-10,000 unique sgRNAs" for better clarity (L674)

"Next, sgRNAs with a 10-fold difference in enrichment factor between replicates were removed."
"≥ 10-fold"?
Yes, modified in the text (L679)

For all dot plots, please consider adding transparency to better visualize superimposed individual dots as in Fig. 2c. Concerned figures are Fig. 3a, 4b, 5a, as well as Supp. Fig. 3a, 4a, and most importantly Supp. Fig 10
Transparency was added to the dot plots in the respective figures.

Reviewer #2 (Remarks to the Author):

Review on manuscript NCOMMS-19-35755
"Pooled CRISPRi screening of the cyanobacterium Synechocystis sp. PCC 6803 for enhanced growth, tolerance, and chemical production"

Cyanobacteria play important roles in many aquatic and terrestrial environments, as primary but also as toxin producers. In addition to their global ecological role, cyanobacteria recently came also in the focus as green cell factories to establish CO2-neutral biosynthesis of different products. Despite many promising examples for proof of concept, the production titers are mostly low, which is certainly related to our lack of knowledge on cyanobacterial regulation mechanisms for growth and for metabolism. The CRISPRi technique is a powerful tool to target many or even all genes in a bacterium and subsequently analyze the effects on different cellular levels. The authors showed here the genome-wide CRISPRi application towards the model cyanobacterium Synechocystis sp. PCC6803, which is often used in the basic and applied research of many different aspects among cyanobacteria. The authors screened the CRISPRi libraray for genes potentially limiting growth of Synechocystis or have
impact on L-lactate production with this strain. They present lists of genes that were depleted of enriched in the sgRNA mutant library under different growth conditions. In some cases, they started also attempts to verify the role of identified genes by single CRISPR-induced gene silencing approaches. These approaches showed that the initially identified genes seem to play a role in the investigated traits, but more work is necessary.

Major points

1. The real efficiency of the CRISPRi attempt is not really clear. The authors mention that the obtained 300,000 Synechocystis transformants, which were pooled and used for the later experiments. Please show data, how many of the initially synthesized sgRNAs were really included in the libraries, which were used for the three different applications. Did you observe cases that more than one sgRNA construct was found in one Synechocystis clone?

We acknowledge that key numbers of the sgRNA library were not sufficiently clearly presented. We added the following information to the beginning of the Results (L81):

"We designed a CRISPRi library for repression of each annotated gene in *Synechocystis* (3546 ORFs, 1871 ncRNAs)[16]. We designed two sgRNAs for each of 3526 ORFs and 1555 ncRNAs, and one sgRNA for 20 ORFs and 316 ncRNAs, totalling 10498 sgRNAs*."*

The number of actually NGS-quantified sgRNAs for each competition experiment and time point is shown in Suppl. Figure 11 b. With a few exceptions, around 6500 to 7000 sgRNAs per condition were quantified. The obtained median read count per condition and time point is shown in Suppl. Figure 11 a.

This does not include the sgRNA library in the lactate overproducing strain which is a separate library. We added text stating the required information to the results sections (L262).

Regarding two sgRNA constructs in one cell. Before subjecting the library to NGS, we sequenced the sgRNA region of 12 clones using Sanger sequencing. From these, all had only 1 sgRNA in the genome insertion region. Unfortunately we cannot say for certain from the NGS data that all clones have only 1 sgRNA, since the sequenced fragments are short (75 bp).

However, while it may be possible for more than 1 sgRNA construct to enter the cyanobacteria cell, the transformation efficiency is so low (approximately 10,000 per ug DNA, or 3-4 orders of magnitude less than of E. coli), that it is extremely unlikely.

It is also unlikely that two protospacers from the library could be cloned into the same vector, since cloning of the protospacer region was directional (GoldenGate cloning).

2. The authors are very confident. I think the CRISPRi screening only identifies genes that are potentially/possibly related to growth limitation or lactate productivity. Enrichment or depletion could also result from indirect effects. Therefore, I would suggest to weaken the statements and to improve discussion on advantages but also problems with this approach.
It is correct that only growth-related phenotypes will be visible in our competition experiments. Phenotypes with altered metabolism but neutral effect on growth rate can not be identified. The strategy of screening a CRISPRi library can therefore mostly identify true positives while the rate of false-negative observations can not be determined. We are nevertheless confident that it is powerful in generating hypotheses i.e. finding candidate genes for certain cell functions. However, the actual mechanism of a candidate gene/protein has to be validated in a second step. We expanded the paragraph about limitations of the CRISPRi approach (see L410 in Discussion).

Specific points:

1. Second line abstract: The data presented in the manuscript clearly show that not all genes were really targeted, please mention that you <u>aimed</u> to target all genes!
The reviewer is correct in the sense that not all genes were equally well repressed due to CRISPRi limitations. The abstract now reads: "we aimed to target all genes". We think that this statement is still accurate because every known ORF was targeted with 1 or 2 sgRNAs, although this does not automatically mean efficient repression.

2. Second last sentence of introduction: What means “biochemical production”?
Changed to "increased productivity of a target compound"

3. End of first para Results: As mentioned above, it is essential to show the data how representative your library was.
We have now included more results about the depth of coverage of all libraries in Methods.

4. Second para: please mention the light or dark periods, e.g. 12/12 h
The information was included in the introduction and results section (L94).

5. Second para: 7119 sgRNA clones were investigated. This corresponds to about 60% of the initial library, what does this mean, did you found these 7119 sgRNAs clones in each treatment, i.e. are in each treatment always the same sgRNAs missing?

We apologize for the inaccurate wording. The 7119 sgRNAs (now corrected to 7072 sgRNAs, because some erroneous sgRNAs were removed) are all the sgRNAs targeting ORFs, while all others target non-coding RNAs. Of the 7072 ORF-targeting sgRNAs, the majority (5590) were quantified in all 39 samples. The coverage of each sgRNA versus number of samples where it was quantified is shown below. The phrase was corrected to: "The abundance of the 7072 sgRNAs (clones) targeting ORFs were quantified and averaged across the 4 replicates"*. (L99 and also L82)*

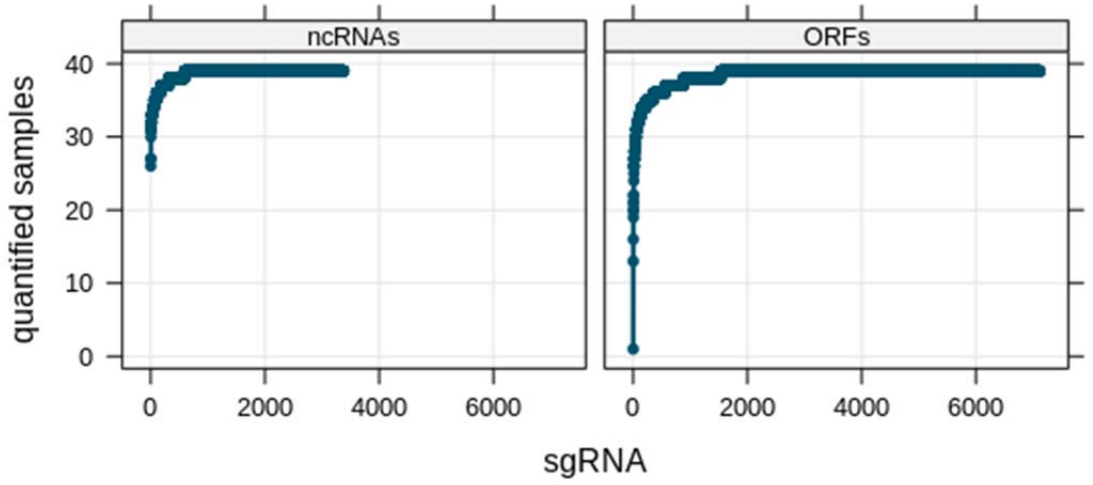

6. Second para: summing up cluster 1-4 results in 1992 and not as mentioned above 1998

The reviewer is correct. The number of 880 sgRNAs for cluster 3 as written in the results section was wrong, there were 886 sgRNAs (then summing up to 1998 sgRNAs for clusters 1 to 4). We redid the clustering analysis after some sgRNAs were removed and cluster assignments have now changed slightly. All sgRNA numbers per cluster 1-4 now add up to 1998 (241 + 378 + 767 + 612).

7. Second para: summing up clusters 1-5 results in 7113 sgRNAs and not 7119 mentioned above

Related to the same mistake as above.

8. End of second para: how good is the overlap to essential genes identified in the transposon-based screen in S. elongatus?

Concerning genes related to central carbon metabolism, there is a 75% agreement between high fitness genes (i.e. at least one sgRNA clone is depleted significantly) identified by our CRISPRi library and essentiality as determined by TN-Seq of S. elongatus. This is now clarified in the Discussion (L 387) and in Supp. Data 10.

9. Page 5, last para: the authors discuss two possibilities why normal light and high light data vary. I suggest a third explanation, which is related to light/oxidative stress induction in the

high light cultures. These cells are probably already more stressed than cells at 100 micro Einstein leading to the severe down-regulation of key enzymes? This assumption is well supported by genes found in cluster 3, many of them known to be crucial for high light/redox stress acclimation.
We added this hypothesis to this paragraph (L148). We did not consider that cells could experience light stress already at 300 µmol photons m^{-2} s^{-1}, which is the intensity in our cultivation setup where cells reach maximum growth rate. Synechocystis continues to grow with high rate at up to 1000 µmol photons. However, it is quite possible that continuous light induces stress even at a 'moderate' intensity (such as 300 µmol photons) where it is not causing photo-inhibition in regard to growth rate.

10. Page 5, last sentence: The lower effect of L/D growth on essential genes could be also related to the fact that S. elongatus has a much higher percentage of strictly circadian-regulated genes than 6803?
We thank the reviewer for the suggestion and included this explanation (L165).

11. Page 6: The finding of omega subunit is remarkable, since the mutant is impaired for example in carbon acclimation. May be that this point could be discussed.
It is indeed surprising that the rpoZ (ssl2982) knock-down mutant showed a growth advantage over the wild type. It was reported by Gunnelius et al. that a Synechocystis PCC 6803 rpoZ deletion mutant is viable and shows no impaired growth compared to the wild type under growth in air (low CO2), either at low light (40 uE) or moderate light (90 uE). In fact, there appears to be a slight growth advantage of the rpoZ mutant in Kurkela et al (Table S1; doi.org/10.1104/pp.16.01953). In our results, we detected a slight growth advantage of ca. 5% above population growth rate, and only for the high light condition.

As the reviewer points out, the same group showed that the rpoZ was defective in growth when low CO2 is switched to high CO2 (i.e. it cannot acclimate to high CO2).However, the assay conditions where we screened the library for growth were different than in Gunnelius et al, in that our cultures were already acclimated to high CO2 (1%) before CRISPRi gene repression was induced. We now discuss these differences in the Discussion section, as well as the observation that an rpoZ mutant may grow faster under constant conditions but is impaired in fluctuating or stress conditions (L348 and L418)

12. Page 7, first para: The upregulation of carotenoid synthesis genes is attributed to PSII repair. Why? Direct evidence for enhanced PSII repair would be expression stimulation of genes for different D1 protein genes or FtsH genes. Probably more carotenoids simply show a general acclimation to light stress.

We thank the reviewer for the interpretation. We have toned down the claim about PSII repair.

13. Page 7, first para: ndhD1 and ndhD2 are not crucial for CO2 uptake but more specifically related to cyclic electron flow!

We now state the importance of ndhD1/D2 for CEF in the text (L215 and L299)

14. Page 7 first para: The differential regulation of rpaB in the selected sgRNA mutants best explains why many genes related to photosynthesis are changed in this mutant set. I would start with this finding and then discuss the other findings in relation to the effect of this or possibly some other regulatory proteins.

We have taken the reviewer suggestion to start discussion of these results with RpaB (L209).

15. Page 7, second para: (up to 15 mM)
Corrected.

16. Page 7, second para: The authors mention that many clones had growth defects in the absence of L-lactate. Please show these data!
The data is shown as grey area in Supplementary Figure 6 (the NaCl control). The L-lactate treatment is shown in color. We added this information to the respective paragraph (L233-234)

17. Page 8, first sentence: The assumption that certain amino acids may have specific protective effects can be easily tested, just by addition of single amino acids to the L-lactate medium, since for most of them uptake systems exist in 6803!

We took the reviewer's advice and screened the effect of added amino acids on lactate tolerance. We chose 3 amino acids representing basic, neutral, and acidic, and where Synechocystis uptake rates were apperciable. We found that addition L-asp and L-gly (10 mM) did not improve growth in the presence of lactate (growth was actually hindered), while addition of L-arginine did. The amino acids were pH-adjusted before addition (Figure below).

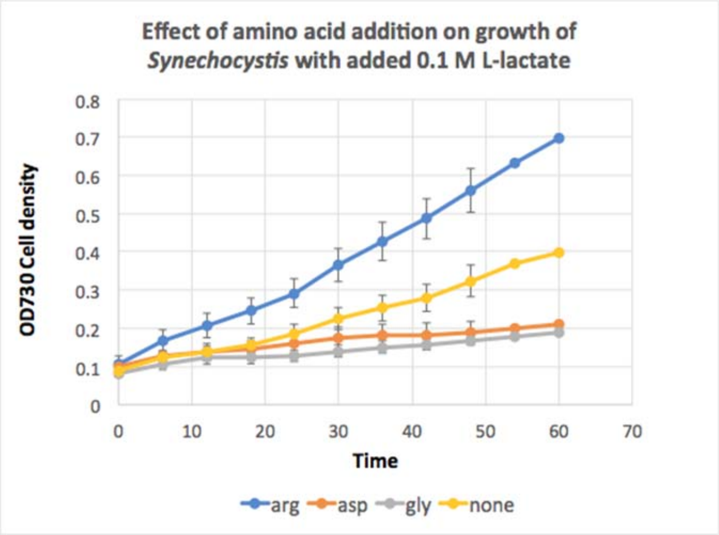

While Synechocystis has a weak amino acid transport capability compared to heterotrophic bacteria (Labarre et al., 1987 DOI: 10.1128/jb.169.10.4668-4673.1987), these follow up

experiments suggest that the majority of the effect of increased tolerance to L-lactate in the tRNA synthetase repression mutants is likely due to the general growth rate decrease that arises. The observation that the effect is seen for repression of multiple tRNA synthetases spanning positive, neutral, and basic amino acids, suggests the effect is not amino-acid specific.

We have therefore removed reference to an amino-acid specific effect in the text and have not included these amino-acid addition experiments so as not to digress too much from the main story of the paper.

18. Supp.Fig. 7: I did not see growth improvement for all repression clones in Suppl. Fig. 7, please show delta growth of repression strains minus growth of control mutant. The growth oscillations are very surprising. After my opinion the oscillation are not very likely due to circadian rhythm. For Synechocystis never long free-running rhythms have been shown! May be that the rhythm was induced by lactate additions? it is also not obvious for all growth curves!
It is true that not all strains showed a clear growth advantage when comparing single knockdown mutants. For example, repression of asd didn't show any and several other knockdowns showed only a non-significant increase. However, the average growth rate shown in Figure 5C was directly determined from the growth curves shown in Suppl. Figure 7, and was in most cases higher than the control, mainly due to higher growth rate at the onset of the cultivation, or weaker oscillation. The growth rate oscillation of Synechocystis even under constant light is an often observed phenomenon in our lab. It was also reported previously by other labs e.g. Van Alphen & Hellingwerf, PLoS ONE, 2015 http://dx.plos.org/10.1371/journal.pone.0127715). Figure 1 in the referenced publication shows oscillation under constant light for at least 5 days.

19. Fig. 5: if I understood the symbols in the figure right, only two repression clones show significantly improved growth! or is the growth of all light grey columns significantly improved by $p = 0.05$? This is unlikely at least for the asd gene. Please refer only to genes that were verified by statistical significant differences.
Yes, only two clones showed a significant growth advantage according to student's t-test and a significance threshold of $\alpha \leq 0.05$, as indicated by star symbols. We changed the colors of the bars to all white in order to avoid confusion.

20. Page 8, end of first para: After my opinion, the improved pigmentation simply shows less toxic effects of L-lactate.
We have removed claims about increased photosynthetic activity

21. Page 8, last para: again numbers are missing, how representative the library really was!
We have now included data about the depth of the library in the lactate-producing strain (all sgRNAs were present; L261

22. Page 9, first para: The authors refer to genes that were found in two out of four attempts. Is that statistical significant? how many genes were reproducibly found in three or even four sorted samples?

We used the criterium that a sgRNA should be enriched in 2/4 runs to narrow down a potential list of sgRNAs to validate as single clones. We found 23 sgRNAs enriched in 3/4 sorted samples, and 1 sgRNA enriched in all 4 sorted samples. This is now explicitly stated in the text (L279-284). Despite technical variability, we are confident that this method could prove to be helpful to find mutants with improved phenotypes, as we found many targets in the list that were predicted computationally, .

23. Page 9, middle para: The ATP/NADPH ratio is discussed as main possibility to improve production of certain biochemical. Then, two sgRNA clones are mentioned that possibly have enhanced ATP levels. Did you try to measure the ATP amounts to support this speculation?

The reviewer is correct that we predict slr2016 and sdhA mutants to have lowered ATP/NADPH ratios. We did not measure ATP or ATP/NADPH ratios in any of the reconstructed clones. These metabolites are known to vary widely with growth rate so that measurements are difficult to compare across cultivations (Asplund-Samuelsson et al., 2018). However, after this paper was submitted, a laboratory at Turku measured ATP/NADPH in a Synechocystis flv3 mutant, which was predicted by FBA to increase the ATP/NADPH ratio. Flv3 was among our enriched clones in the lactate sorting (2/4 runs). They found that the ATP/NADPH ratio decreased by 20% compared to wild-type (Thiele et al 2019, https://doi.org/10.1186/s12934-019-1238-2). These findings give us some confidence in the model predictions that ATP/NADPH would be increased in other mutants even though we do not explicitly measure them. We now also describe the results from the Flv3 mutant in Results (Line 299-301).

24. Table 1: I would expect at least some overlap with genes that were identified in the screening for L-lactate resistant clones, why is there nothing?

We think that the absence of targets related to lactate tolerance in the lactate productivity data is linked to the setup of the experiment. We leave cyanobacteria in droplets to produce lactate for 4 hours so that the lactate in droplet remains in the dynamic range of the lactate assay (1-10 μM). The tolerance to lactate is on the order of mM. Therefore, we believe that the amount of lactate produced in droplets is much lower than the tolerance limit, so we would not get targets related to lactate tolerance in the lactate productivity data.

25. Figure 6: from the data in Fig. 6 it is not clear which differences at the last sampling point are statistically significant, please include this information!

We performed Student's t-test in order to test significance. Only gltA and *pcnb (this target was not included in first submission) showed significance with p <= 0.05 (Figure 6 a). The statistical test was included in the figure and caption. We now also indicate in the text if strains gave significance different lactate production.*

26. Discussion, third line: how cyanobacteria POTENTIALLY solve…

We softened our claim.

27. Page 11, end: 87% overlap is mentioned to FBA, this overlap is surprisingly high, since the model approach can only include annotated genes, whereas here often genes were found coding for proteins of unknown or hypothetical proteins, which are certainly not in the FBA. In the clusters mentioned above about 1900 genes were involved, where the number 329 comes from?
The reviewer is correct that FBA will undercount the number of essential genes. We are referring to the fraction of those 329 FBA-predicted essential genes that we also find as "essential" (strong fitness effect) in our library. The number '329' is the number of essential genes obtained using Flux Balance Analysis of iJN678 (Nogales et al., 2012). We also recounted with padj < 0.005 as threshold. Now 86% of this gene set had at least one (of the two) sgRNA clone depleted in L300 experiment. We modified our statement to ensure more clarity. (L382)

28. Fig. 6a: colors difficult to distinguish
Colors were changed to improve readability.

Reviewer #3 (Remarks to the Author):

Review for Yao2019 NCOMMS-19-35755

- First time genome-scale essentiality data. Important and useful technological achievement. Identified several "mutants" with enhanced biotechnological properties, several of which were also verified independently by reconstruction.

- Of major concern is the lack of accessibility in the figures, in particular. Not only are there too many unneccessary abbreviations, it is often impossible to understand what the point of any one figure panel from the figure itself, even when reading the legend.

We appreciate the reviewer's perspective. We have edited several figures in the text for clarity, and tried to reduce abbreviations.

- The second concern is the apparent lack of data from diurnal conditions even though some early experiments did include such conditons - why not report on the data from them as surely it must have been collected?

The reviewer is correct that we did collect data from di-urnal experiments (and all data is available in the online Shiny application). We do not focus on this data much in the manuscript since the calculated gene fitness scores were similar to those collected in the high-light condition. We have now added more text in Results from the di-urnal experiments, where we state that only a few genes showed differential fitness in LD compared to L300 (there were only 5 genes), and that these were related to CO2 uptake. We also speculate that this small number (compared to Synechococcus) is due to the larger gene set in Synechocystis (potential redundancy), and that Synechocystis appears to have far fewer circadian oscillatory genes than Synechococcus. (L158-166)

- The verification of the mutants with improved growth rate is unclear or missing (difficult work out which)?

We did test the faster-growing mutants from Figure 4 in batch culture. For pmgA, slr1916, sll1969, and ssl2982, the increase in maximum growth rate (measured at early log-phase) was confirmed to be 8-15% higher than wild-type. However, for slr1340, the growth rate measured in batch was not higher than wild-type. However, we decided to leave the RNA-Seq of slr1340 in, since this strain did show enrichment over the course of the library competition experiment. Overall, the growth-rate differences for this strain would be small, so that calculation of μ_{max} from batch cultures may mask differences. We have added results of the batch-phase validation experiments as new Supplemental Figure S6:

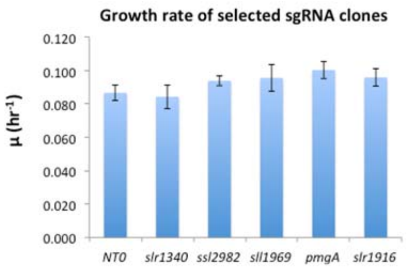

Strain	u (hr^{-1})	Stdev	p-value
NT0	0.087	0.005	--
slr1340	0.085	0.003	*n.s.*
ssl2982	0.094	0.005	0.041*
sll1969	0.096	0.008	0.012*
pmgA	0.100	0.007	0.003**
slr1916	0.098	0.01	0.013*

- Overally, the writing is often sloppy without consideration of intellectual interest for the reader. The data is actually quite interesting and there is more to discuss than presented. In addition, there is considerable discussion in the results section (e.g. page 6), why not just combine the two?

We have taken efforts to clean up the writing throughout the text. We moved some of the discussion present in the Results to the Discussion section instead. In the end, we decided against combining Results and Discussion for the sake of clarity.

Improvements
- Line numbers are essential for an effective review process

We agree and have now added line numbers in the latest submission

- Abstract is unclear, 3rd to last sentence. 2nd to last sentence is even more unclear, perhaps due to a word limit? What caused what? % what?

We have now edited the abstract for clarity. The 150 word limit on the abstract restricts us in how descriptive we can be.

- Introduction, the need in general by the research field is not specifically essentiality, but rather better understanding on how the system works as a whole. Essentiality is part of that but not the only knowledge we need. How many genes in 6803 have a verified function relative to E. coli, for example? Some context would be useful.

We have taken the reviewer's advice and tweaked the introductory text to point out that around 45% of genes in model cyanobacteria have no known function. We have also noted that screening of gene essentiality (or fitness) in multiple conditions is one avenue to elucidating gene function. (L34, L41)

- 'both photoautotrophic and diurnal growth' is unclear? When is the latter not also the former?

This was a mistake, we have changed the text to read "both constant light and diurnal growth" (L44)

- Does '>10,000' = 'all annotated genes' = 'all open reading frames'? The start of the Results section (no line numbers provided) suggest they are not the same? How many genes are annotated in 6803 and where was that done, i.e. on what basis?

We now state in the Introduction that the sgRNAs in our library target protein coding genes as well as non-coding RNAs. We also specify in the Methods section that the annotations for ORFs were taken from NCBI, and the annotations for ncRNAs were from experimental reports. (L81-L83)

- Similarly, 2 x each gene => 10,498 sgRNA sequences => Less than 10,000 genes???

This is now clarified in the Results section, where we state that most but not all ORFs and non-coding RNAs are targeted by 2 sgRNAs. Of a total of 10,498 sgRNAs, 7072 sgRNAs are targeting ORFs (3526 with 2 sgRNAs and 20 with 1 sgRNA) and 3426 sgRNAs are targeting ncRNAs (1555 with 2 sgRNAs and 316 with 1). (L81-L83)

- 'several knockdowns.'feels sloppy'
The sentence now reads: We show that there are multiple gene knockdowns that increase the growth rate of Synechocystis. (L66)

- Overall, the introduction feels like a collection of separate items rather than a smooth-flowing story. Why is this work of interest other than just technological achievement?

We have added some text in the Introduction to emphasize how the CRISPRi library characterized here will be important for the field. We suggest screens that could give more insight into cyanobacteria metabolism (such as whether genes that increase growth speed reduce robustness).

- Last sentence in first paragraph of Results section is poorly written.
We have reworded this sentence for clarity. (L87)
"The resulting Synechocystis clones were pooled and constituted the CRISPRi library. Deep sequencing of the sgRNA region of the library confirmed that all of the designed 10498 sgRNAs were present."

- Was there any contamination of the cultures? Was this checked for in any way?
Contamination was checked at the end of each cultivation by plating cultures on LB agar. No contamination was observed. Contamination was unlikely, as antibiotic selection was used throughout.

- What is 'depletion pattern'?
We have clarified that depletion pattern is the rate of washout from the turbidostat. (L101)

- 'We examined genes in central carbon metabolism for effects of CRISPRi repression'. Why? How?
We have clarified the purpose of this paragraph: that our fitness data can be used as support (or against) current model-based predictions of reaction fluxes in Synechocystis. (L123)

- 'A second explanation is that faster growing cells are more prone to perturbation of enzyme levels, due to higher enzyme saturation' is unclear
We have removed this sentence

- First paragraph in 'mutants with a growth advantage'. What was the outcome from diurnal conditions? For example, was the outcome similar for calvin cycle and photorespiration related genes? That would be more interesting in relation to fundamental biology than constant light.
The fitness scores for CBB cycle genes and photorespiration genes were similar in constant light and in the di-urnal condition (this is alluded to in a previous section). More specifically, our calculation for fitness score for these genes in these two conditions did not meet p-value criteria for significance. However, three genes related to the carbon-concentrating mechanism (CCM) had differential fitness scores. Ndh3 and slr1302 (CO_2 hydration protein family) are both associated with CO_2 uptake and hydration by the NDH-1 complex. Both genes were found to be essential for growth in constant light but dispensable in LD conditions. Conversely, repression of the carboxysome structural gene ccmM reduced growth more in LD conditions than in LL conditions. This is now stated in the Results (L159).

Also, the mutants pmgA and slr1916 mutants showed increased growth in constant light and in diurnal conditions. This is now indicated in the Results (L176).

- Were the mutants with increased specific growth rate confirmed separately as was done with the mutants identified to have improved growth in the presence of lactate? I was not able to see this and the conclusion on this part of the work was muddy.

We did test the faster-growing mutants from Figure 4 in batch culture. For pmgA, slr1916, sll1969, and ssl2982, the increase in maximum growth rate (measured at early log-phase) was confirmed to be 8-15% higher than wild-type. However, for slr1340, the growth rate measured in batch was not higher than wild-type. However, we decided to leave the RNA-Seq of slr1340 in, since this strain did show enrichment over the course of the library competition experiment. Overall, the growth-rate differences for this strain would be small, so that calculation of μ_{max} from batch cultures may mask differences. We have added results of the batch-phase validation experiments as new Supplemental Figure S6.

- Discussion. How conditional is the outcome? What if the expression of the sgRNA varied, for example? Surely this must have been optimized at some point of the study?

As the reviewer suggests, the outcomes reported here will be dependent on the strength of transcriptional repression, which itself is dependent on the abundance of dCas9, the sgRNA sequence, and the region of gene that is targeted. With this particular promoter, and by targeting the sgRNA to the 5' end of the gene, we typically achieve >90% repression of the target gene. However, in a small fraction of cases, we have observed in some cases repression efficiency of 50% (reference to Yao et al., 2015, Kaczmarzyk et al., 2018). Therefore, throughout the paper we have focused on gene targets where both designed sgRNAs show a growth effect. The discussion has been modified to address this point. (L380)

- How can we interpret this data in the context of evolution? The conditions were highly specific, yet evolution most likely has favoured robustness over specific growth rate speed? It would be interesting to see some discussion on this even if speculative.

The reviewer raises a very interesting point. Though we did not screen for robustness here, such tests are definitely possible with the CRISPRi library and are indeed a unique application of it. It may be that some of our faster-growing clones are not tolerant to, for example, fluctuation in light intensity or substrate availability, and vice versa; slower growing mutants in constant conditions may have a fitness benefit in fluctuating conditions. Such comparisons could be addressed in future work. We have added some text about this in the Discussion (L416 and following).

- Figures. There is no need to abbreviate everything. FC, dF, F, it's too much. Spell it out instead. Similar for NACL and LAC, why not use much more recognisable words? NaCl, or Lactate?
We followed the reviewers suggestion and spelled out abbreviations where possible.

- The majority of the figure panels are impossible to follow though I imagine a specialist working with similar methods will understand. Should the results be accessible to all readers or only some?
We tried to present the results in an appealing and comprehensive fashion. We also tried to make symbols and colors consistent throughout (sub-) figures in order to provide some guidance. However, we also addressed criticism of the reviewers and hope that figures are now more comprehensible in our re-submitted manuscript.

4C, what do the colours mean? There is no explanation in the panel. There is space to describe it in the figure rather than just in the legend.
We added the missing legend title to Figure 4 C ('log_2 fold change').

4D what is the relationship between the genes written over the grey background at the top of each sub-section and those within the rectangles?

Figure 4 presents the results of a transcriptome analysis of four selected CRISPRi knockdown strains. The four panels in sub-figures 4 A,B and D represent one mutant each. The dots in 4D represent up or down-regulated genes with fold change on X axis and functional category on the Y axis. We tried to make this more clear in the figure caption.

5E show the difference to wild-type instead, a large number of very similar spectra with very similar colours are practically impossible to decipher.
We agree that this sub-figure was hard to read. We have reduced the number of overlaid spectra that are shown. All subfigures 5 d, e, and f now present the same set of the three fastest growing sgRNA mutants.

Fig. 6 would benefit from statistical analyses in the figure rather than just error bars.
Figure 6 was improved by adding the p-value / symbols for a Student's t-test.

REVIEWERS' COMMENTS:

Reviewer #1 (Remarks to the Author):

The authors have satisfactorily addressed all my concerns. Congrats on a nice piece of work!

Reviewer #2 (Remarks to the Author):

none

Reviewer #3 (Remarks to the Author):

The manuscript is improved. The figures are still challenging to understand, but that is a subjective matter. I would recommend that the writing should consistently (also abstract) be in past tense unless the research is still ongoing.

REVIEWERS' COMMENTS:

Reviewer #1 (Remarks to the Author):

The authors have satisfactorily addressed all my concerns. Congrats on a nice piece of work!

Reviewer #2 (Remarks to the Author):

none

Reviewer #3 (Remarks to the Author):

The manuscript is improved. The figures are still challenging to understand, but that is a subjective matter. I would recommend that the writing should consistently (also abstract) be in past tense unless the research is still ongoing.

We have not changed the tense of the abstract as per journal requirements
